# CD31 defines a subpopulation of human adipose-derived regenerative cells with potent angiogenic effects

**DOI:** 10.1038/s41598-023-41535-1

**Published:** 2023-09-01

**Authors:** Pratibha Dhumale, Jakob Vennike Nielsen, Anne Cathrine Schmidt Hansen, Mark Burton, Hans Christian Beck, Mads Gustaf Jørgensen, Navid Mohamadpour Toyserkani, Martha Kirstine Haahr, Sabrina Toft Hansen, Lars Lund, Mads Thomassen, Jens Ahm Sørensen, Ditte Caroline Andersen, Charlotte Harken Jensen, Søren Paludan Sheikh

**Affiliations:** 1 Department of Clinical Research, University of Southern Denmark (SDU), Odense, Denmark; 2 Department of Clinical Biochemistry, Odense University Hospital (OUH), Odense, Denmark; 3 Department of Clinical Genetics, OUH, Odense, Denmark; 4 Department of Plastic Surgery, OUH, Odense, Denmark; 5 Research Unit for Plastic Surgery, Department of Clinical Research, SDU, Odense, Denmark; 6 Department of Urology, OUH, Odense, Denmark

**Keywords:** Stem cells, Diseases, Urology

## Abstract

Cellular heterogeneity represents a major challenge for regenerative treatment using freshly isolated Adipose Derived Regenerative Cells (ADRCs). Emerging data suggest superior efficacy of ADRCs as compared to the ex vivo expanded and more homogeneous ADRCs (= ASCs) for indications involving (micro)vascular deficiency, however, it remains unknown which ADRC cell subtypes account for the improvement. Surprisingly, we found regarding erectile dysfunction (ED) that the number of injected CD31+  ADRCs correlated positively with erectile function 12 months after one bolus of autologous ADRCs. Comprehensive in vitro and ex vivo analyses confirmed superior pro-angiogenic and paracrine effects of human CD31+ enriched ADRCs compared to the corresponding CD31− and parent ADRCs. When CD31+, CD31− and ADRCs were co-cultured in aortic ring- and corpus cavernous tube formation assays, the CD31+  ADRCs induced significantly higher tube development. This effect was corroborated using conditioned medium (CM), while quantitative mass spectrometric analysis suggested that this is likely explained by secretory pro-angiogenic proteins including DKK3, ANGPT2, ANAX2 and VIM, all enriched in CD31+  ADRC CM. Single-cell RNA sequencing showed that transcripts of the upregulated and secreted proteins were present in 9 endothelial ADRC subsets including endothelial progenitor cells in the heterogenous non-cultured ADRCs. Our data suggest that the vascular benefit of using ADRCs in regenerative medicine is dictated by CD31+  ADRCs.

## Introduction

Adipose tissue is an easily accessible and abundant source of stem cells for regenerative medical therapies and several clinical and preclinical studies have shown regenerative effects of freshly isolated autologous Adipose Derived Regenerative Cells (ADRCs)^1–7^. The heterogenous ADRCs [also known as the stromal vascular fraction (SVF)] consists of many cell types, including pre-adipocytes, stem cells, endothelial cells, various progenitor and immune cells among others^8^. From ADRCs, a plastic-adherent subpopulation can be ex vivo expanded giving rise to a homogenous population of adipose tissue-derived mesenchymal stromal cells (ASCs) that have also been used in many trials with positive outcomes^1,9–11^. ASCs and ADRCs differ substantially in surface protein expression and intracellular cargo which may explain why ASCs and ADRCs perform equally well in some models but not in others^7,12–16^. These differences cannot simply be ascribed to ASC ancestors representing only a minor ADRC fraction (2–10%), since the original ASCs and their descendants reveal important functional and molecular differences within few days of culturing^17^. Whether ADRC sub-fractionation or culture-induced changes are clinically beneficial will likely depend on the context/disease, the route of administration and homing requirements. Indeed, strategies to improve homing of mesenchymal stromal cells (MSC) such as ASCs by increasing their response to injury-induced chemokines and/or to adhere to endothelial cells via selectins could modulate their clinical efficacy^18,19^. Enhancement strategies for homing and function include cytokine-, hypoxia- and scaffold priming as well as molecular and genetic modifications^18,20–22^. Specifically, enhancement strategies aim to increase the pro-angiogenic actions^23–25^. Thus, angiogenesis plays a crucial role for a successful regenerative process. However, despite that ADRCs and ASCs possess the ability to differentiate towards vascular lineages and exert pro-angiogenic effects by releasing secretory factors^26–29^, several studies show that the in vivo regenerative- and the angiogenic properties of freshly isolated autologous ADRCs, are superior to ASCs^15,16,30,31^. This notion is supported by the positive outcomes in several clinical trials using ADRCs, including our own^2,3,6,32^ (reviewed in^1,33^) suggesting that a highly potent vascular subpopulation in the heterogenous ADRCs could be lost during cultivation by the less potent ASCs. Erectile dysfunction (ED) often contains an element of vascular malfunction^34^, and we have previously observed that autologous ADRCs could correct ED, i.e. 8 out of 15 urine continent men regained their erectile function after prostatectomy^2,3^. Since the ADRC properties vary significantly depending on the fat depot as well the age, body mass index, gender, and disease state of the patient^35^, it is likely that non-responders in the ED trial may be explained by some of these variables or the number of pro-angiogenic ADRCs. It will thus be important to define ADRC subtypes that support angiogenesis to improve ADRC based vascular repair in diseases with an ischemic vascular etiology.

We herein investigated endothelial-like ADRCs marked by the membrane protein CD31 (encoded by *PECAM1*) to address their potential in vascular tissue recovery.

## Results

### CD31+  ADRCs cell numbers correlate with patient outcome in a phase I clinical trial of erectile dysfunction

**Figure 1:**
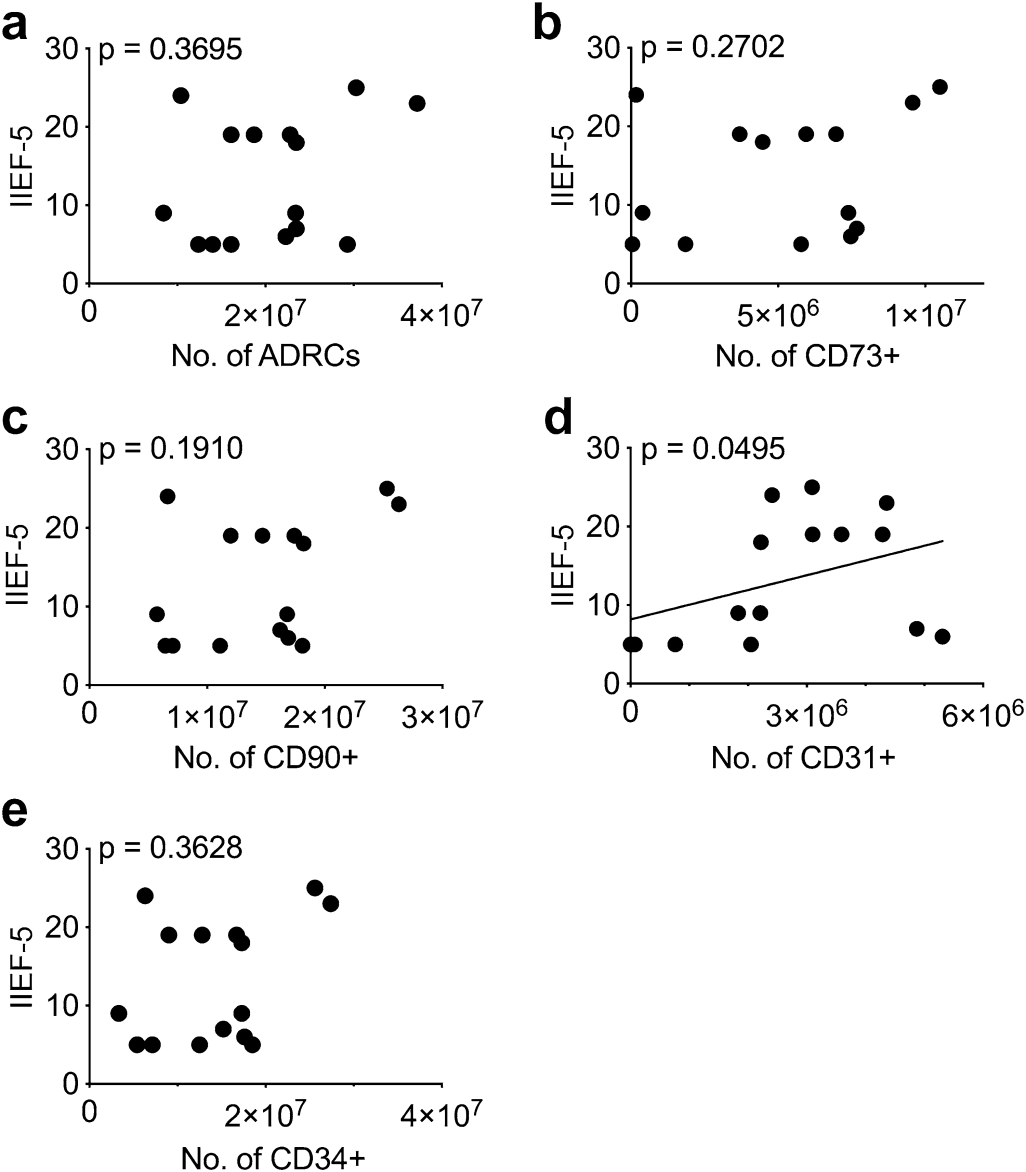
Patient-reported outcomes following cell therapeutic treatment for radical prostatectomy-related erectile dysfunction, correlate positively with the number of injected CD31+  ADRCs. Fifteen urine-continent patients suffering from erectile dysfunction following radical prostatectomy were treated with a single intra-cavernousal injection of autologous ADRCs and the patient-related outcomes (i.e., recovery of erectile function) were evaluated according to the IIEF-5 score 12-months post-treatment^2^. The individual IIEF-5 scores are plotted against: (**a**) the corresponding total numbers of injected ADRCs and the numbers of the (**b**) CD73+ subpopulation, (**c**) CD90+ subpopulation, (**d**) CD31+  subpopulation as well as (**e**) CD34+ subpopulation of ADRCs, respectively, and analyzed for correlation using Spearman r correlation test. p-values are shown in the figure panels. Only the number of injected CD31+  ADRCs significantly correlated with higher, improved IIEF-5 scores.

We previously conducted a Phase I trial using freshly isolated autologous ADRCs to treat erectile dysfunction (ED) after radical prostatectomy (RP)^2,3^. The study was approved by the Danish National Ethics Committee (no. 37054), and is registered at ClinicalTrials.gov (NCT02240823, Phase 1 Study). As previously reported, 8 of 15 urine continent patients (53%) had significantly improved erectile function, measured by the International Index of Erection Function-5 (IIEF-5 scores)^2^. Herein, we investigated whether the ADRC efficacy correlates with total ADRCs injected or with a yet unidentified potent subpopulation defined by either CD31+  (predominantly endothelial cells), CD34 + (stem/progenitor- and endothelial cells), CD73 + (stromal stem cell subset), or CD90 + (stromal- and stromal stem cells). Correlation analyses (Fig. 1a–e) showed that the number of CD31+  ADRCs significantly correlated with the IIEF-5 scores (Fig. 1d), while there was no correlation with the total ADRC number or with other ADRC subtypes. In the 15 ED patients’ ADRCs, 13.9 ± 8.4% (mean, SD) expressed CD31, which is similar to other cohorts^5,6^. Using absolute CD31+  cell numbers vs. 12-month IIEF15 resulted in a significant correlation p = 0.0495 and r = 0.5195 (spearman). However, using %CD31 vs. 12-month IIEF the result did not reach significance (spearman p = 0.2527, r = 0.3151), suggesting that the absolute CD31 cell number but not the CD31 ratio is important for the effect. The clinical correlation with CD31+  ADRCs suggests that they could be responsible for the therapeutic vascular effect of ADRCs^36^.

### CD31+  ADRCs exhibit enhanced ex vivo paracrine angiogenic potential

We next investigated the pro-angiogenic potential of CD31+  cell populations isolated from ADRCs from 5 donors using magnetic cell sorting (MACS). Their enrichment was validated by flow cytometry and qPCR analyses (Supplementary Fig. 1).

**Figure 2:**
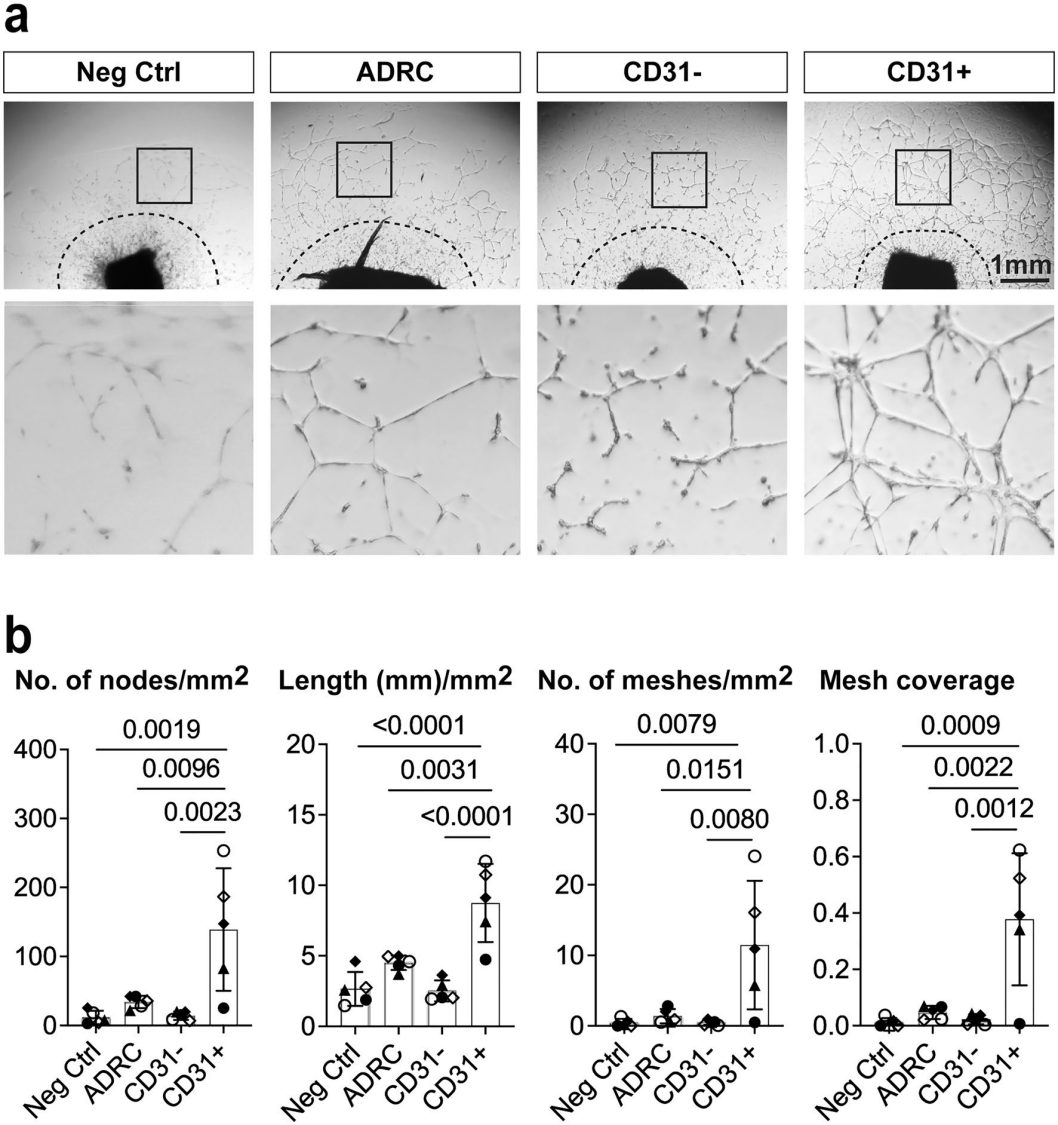
Test of potential paracrine angiogenic effects of human ADRCs, CD31- and CD31+  ADRCs in ex vivo co-culture assays with mouse corpus cavernosum explants. (**a**) Representative pictures of the sprouting from mouse corpus cavernosum explants after 15 days of co-culture with 20,000 ADRCs, CD31−, and CD31+  ADRCs, respectively. Two structurally distinct regions can be identified by visual inspection: (1) in proximity to the corpus cavernosum explants an unstructured area is characterized by high cell numbers but low structural organization; (2) more distally, a well-developed area is characterized with higher structural organization of tubes and establishment of mesh-like structures. The border between these areas is indicated with a stippled line. Quantification of the sprouting was done in three fixed-sized squares, one of these is indicated by a solid-lined box and shown at higher magnification in the lower line of figure panels. (**b**) Quantification and statistical analyses of the sprouting from mouse corpus cavernosum explants, evaluated based on number of nodes/mm^2^, total length (mm)/mm^2^, number of meshes/mm^2^, and mesh coverage i.e., mesh area per area analyzed. Data is based on five experiments, using cells from three male donors and two female donors. Each experiment consisted of triplicates for each tested cell type. Statistical analysis: For each experiment and condition, outliers were identified by the Rout method before normal distribution was confirmed using D’Agostino-Pearson or Kolmogorov–Smirnov normality tests as appropriate. The means of each of the 4 conditions (Negative control, ADRC, CD31− and CD31+  ADRCs) for each experiment were calculated and subsequently compared using one-way ANOVA. Depiction of data: Each data point represents a mean from one experiment. The box represents the mean of 5 means ± standard deviation (SD). Statistically significant p-values are shown in the figure panels.

**Figure 3:**
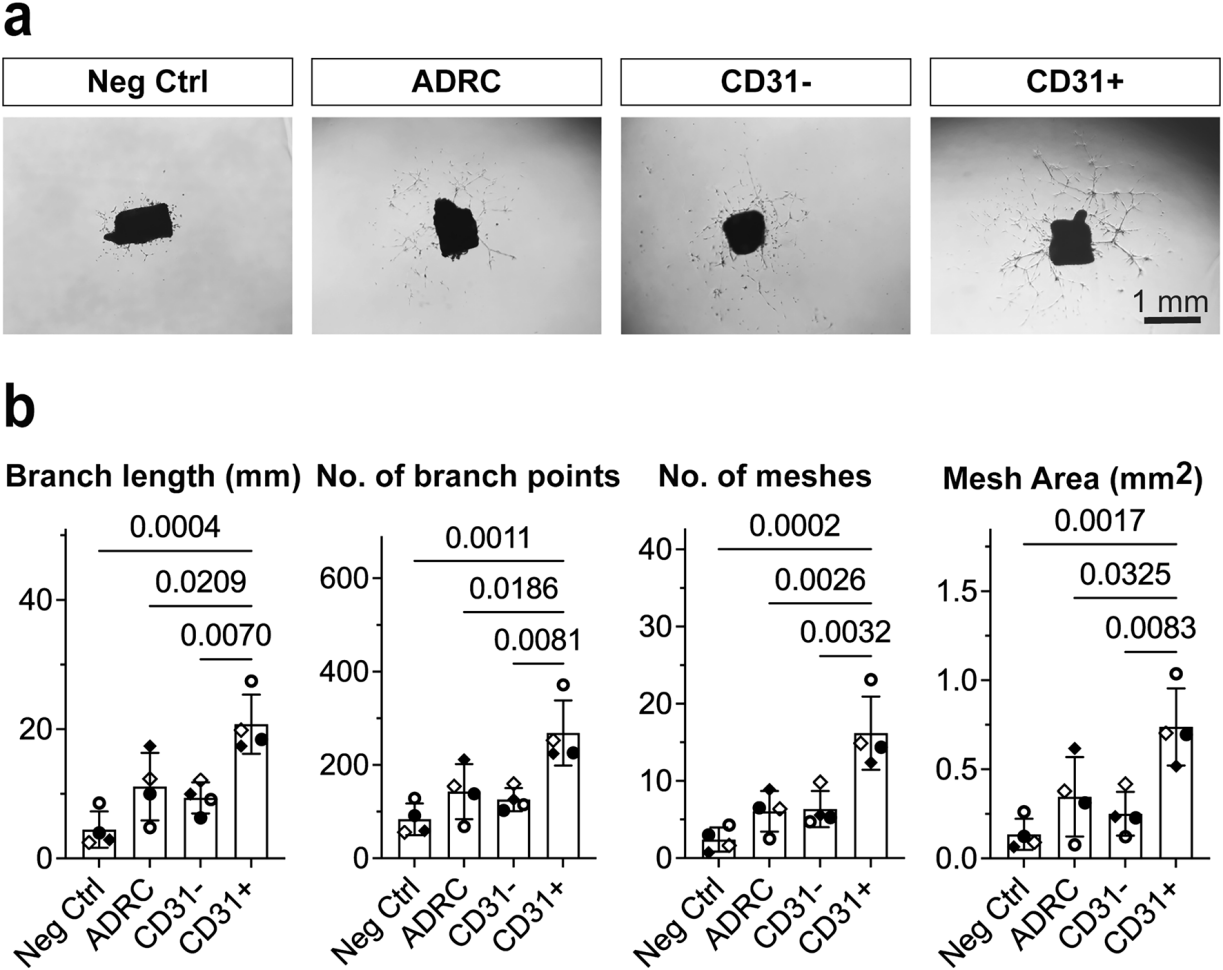
Test of potential paracrine angiogenic effects of human ADRCs, CD31− and CD31+  ADRCs in ex vivo co-culture assays with mouse aortic ring explants. (**a**) Representative pictures of the sprouting from mouse aortic ring explants after 8 days of co-culture with 50,000 ADRCs, CD31− and CD31+  ADRCs, respectively. (**b**) Quantification and statistical analyses of sprouting from aortic rings evaluated based on the total branch length (mm), number of branch points, number of meshes and total mesh area (mm^2^). Data is based on four experiments, using cells from three male donors and one female donor. Each experiment consisted of 6–8 replicates for each tested cell type. For each experiment and condition, outliers were identified by the Rout method before normal distribution was confirmed using D’Agostino–Pearson or Kolmogorov–Smirnov normality tests as appropriate. The means of each of the 4 conditions (Negative control, ADRC, CD31− and CD31+  ADRCs) for each experiment were calculated and subsequently compared using one-way ANOVA. Depiction of data: Each data point represents a mean from one experiment. The box represents the mean of 4 means ± standard deviation (SD). Statistically significant p-values are shown in the figure panels.

We next developed a co-culture ex vivo assay in which human ADRCs were cultured in inserts for 15 days with Matrigel-embedded mouse corpus cavernosum explants at the bottom of the wells (Fig. 2 and Supplementary Fig. 2). The ADRCs initiated explant sprouting at day four, while well-defined mesh-like structures had developed on day 15 (Supplementary Fig. 2b) enabling quantification of sprouting in the well-developed area as previously described for the aortic ring sprouting assay^37^. The CD31+  ADRCs alone elicited a robust and significantly greater sprouting response as compared to ADRCs (p < 0.01, n = 5) or CD31- ADRCs (p < 0.01, n = 5) (Fig. 2), suggesting a paracrine interaction with penile tissue explants. Furthermore, using a standard aortic ring co-culture assay, CD31+  ADRCs again elicited increased tubular formation as compared to ADRCs (p < 0.05; n = 4) or CD31- ADRC populations (p < 0.01; n = 4) (Fig. 3). Sprouting cells from corpora cavernosa stained positively with NG2 but not CD31, (Supplementary Fig. 4a) indicating the presence of pericytes in the capillary-like structures in agreement with previous findings that pericytes are among the first to invade newly vascularized tissue^38,39^. The CD31+  ADRCs superior ability to facilitate angiogenesis and vascular repair increases understanding of the clinical data because of the vascular element in the ED pathogenesis^38,39^.

### CD31+  ADRCs secrete a unique set of proteins associated with angiogenesis

We next looked for differentially secreted proteins by quantitative proteome analysis using mass spectrometry (MS). Conditioned media (CM) from ADRCs, CD31+  and CD31− ADRCs, respectively (n = 3 patients), were analyzed based on the relative protein abundances measured by LC/MS–MS employed with tandem mass tag labeling for relative quantitation. Mass spectra were searched against the Swissport database restricted to humans. Proteins were deduced on basis of at least one unique peptide identified with high confidence (false discovery rate (FDR) < 0.01).

A total of 997 human proteins were detected in all samples, of which 680 were present at high enough concentrations to enable quantification (listed in Supplementary Table 3). Gene ontology (GO) enrichment analysis, using Database for Annotation, Visualization, and Integrated Discovery (DAVID version 6.8; https://david.ncifcrf.gov/), confirmed enrichment of secreted proteins belonging to the extracellular exosome (GO:0070062, FDR = 8.57E−168). A list of GO terms with significantly overrepresentation of detected proteins are given in Supplementary Table 4.

**Figure 4:**
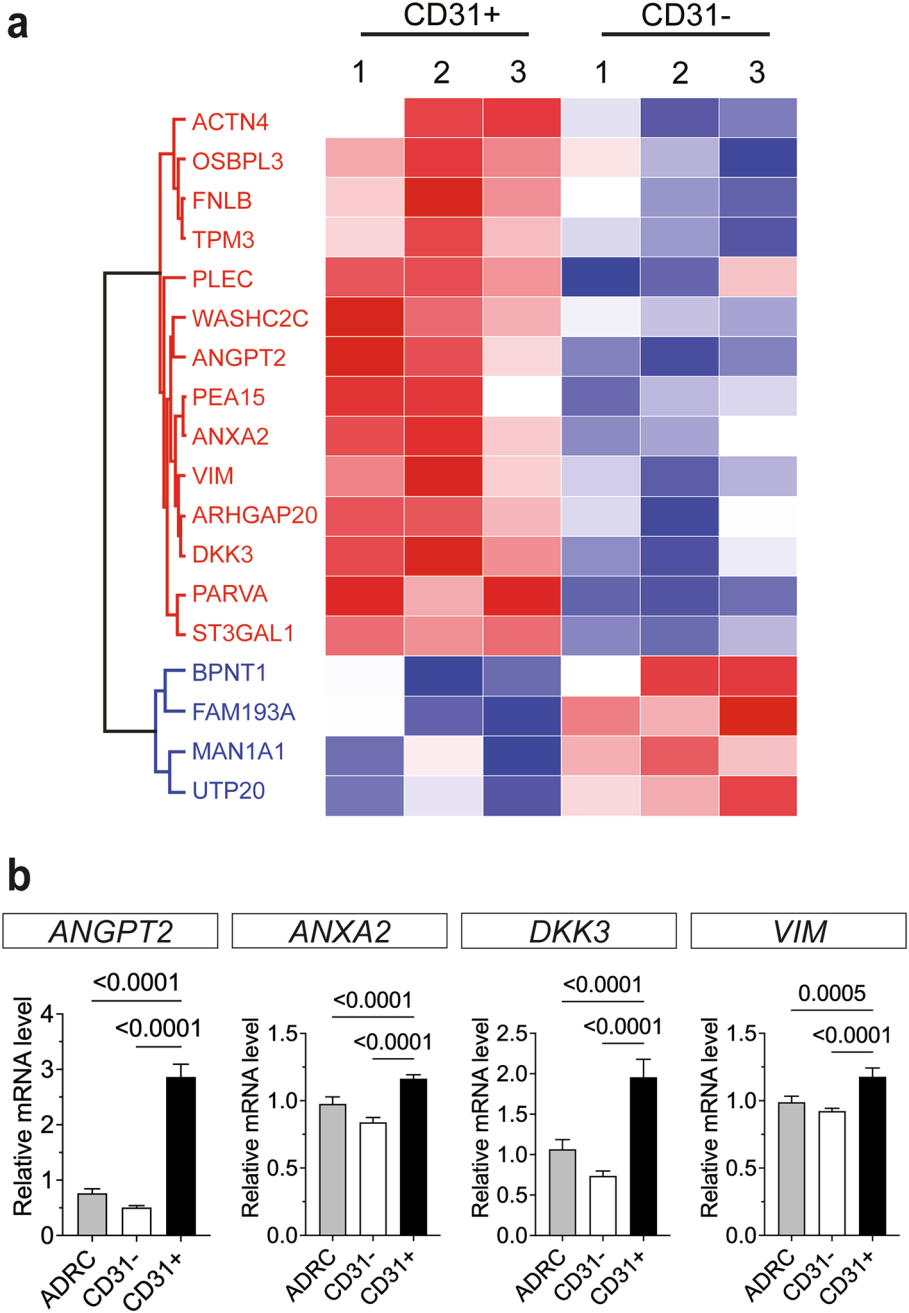
Mass spectrometry reveals significantly different secretomes of CD31+  and CD31− ADRCs. ADRCs, and CD31− and CD31+  ADRCs, respectively, were cultured for 15 days and the isolated proteins from the corresponding conditioned media were analyzed by RP‐nanoLC‐MS/MS analysis. Data is based on cells from three donors (one male and two females). (**a**) Heat map showing 14 significantly upregulated and 4 significantly downregulated proteins in the CD31+  ADRC vs CD31− ADRC conditioned media, as identified by individual t-tests with a p-value of 0.05. Upregulated and downregulated proteins are indicated by red and blue colors, respectively. A volcano-plot-representation of the differentially expressed proteins can be seen in Supplementary Fig. 5. (**b**) Relative mRNA levels of *ANGPT2*, *ANXA2*, *DKK3*, and *VIM* in ADRC, and CD31− and CD31+  ADRCs after 8 days of co-culture with mouse aortic ring explants confirming the upregulation of these transcripts under the conditions in the ex vivo assay. The data was based on cells from one donor, and eight replicates for each of the three populations. To obtain sufficient material, two replicates were pooled in relation to RNA extraction and RT-qPCR performed (in technical triplicates) on the resulting 4 replicates per population. The mRNA expression was normalized to the expression of the reference genes *B2M* and *TBP*, based on the geNorm analysis performed in qBase+ (CV = 0.066, M = 0.191). Statistical analyses were performed using ordinary one-way ANOVA. Statistically significant p-values are shown in the figure panel.

The differential protein expression was further analyzed using the Perseus MaxQuant program. Following hierarchical clustering, we identified eighteen differentially regulated proteins between CD31+ and CD31− ADRC secretomes (Fig. 4a and Supplementary Fig. 5 and Supplementary Table 3). Of these, fourteen proteins appeared nominally upregulated, while four were downregulated in the CD31+ versus CD31− population (p < 0.05) (Fig. 4a and Supplementary Fig. 5). Five known secreted proteins were upregulated in CD31+  ADRC derived CM: Angiopoietin 2 (ANGPT2), Dickkopf 3 (DKK3), Annexin A2 (ANXA2), CMP-N_acetylneyraminate-beta-galactosamide-alpha 2,3-sialytrasferase 1 (ST3GAL1) and Vimentin (VIM). Interestingly, all five proteins have been associated with angiogenesis^40–44^. The expression profiles of all, except *ST3GAL1* were confirmed at the mRNA level in ADRCs, CD31+ and CD31− ADRCs co-cultured with mouse aortic rings (Fig. 4b and Supplementary Fig. 3a). Higher mRNA expression levels of *ANGPT2*, *ANXA2*, *VIM* and *DKK3* as well as *PECAM1* and *VWF*, were also observed in single cultures of CD31+  ADRCs compared to CD31− ADRCs (Supplementary Fig. 3b). This pattern was sustained throughout the culture period that also revealed a notable decline in *ANGPT2, PECAM1* and *VWF* levels between day 8 and day 15 whereas *ANXA2* and *VIM* levels gradually declined as *DKK3* expression increased (Supplementary Fig. 3b). Thus, CD31+  ADRCs secrete a unique set of proteins associated with angiogenesis, which may explain the observed superior vascular effect in vivo.

### Conditioned medium from CD31+  ADRCs induces a robust paracrine angiogenic effect

**Figure 5:**
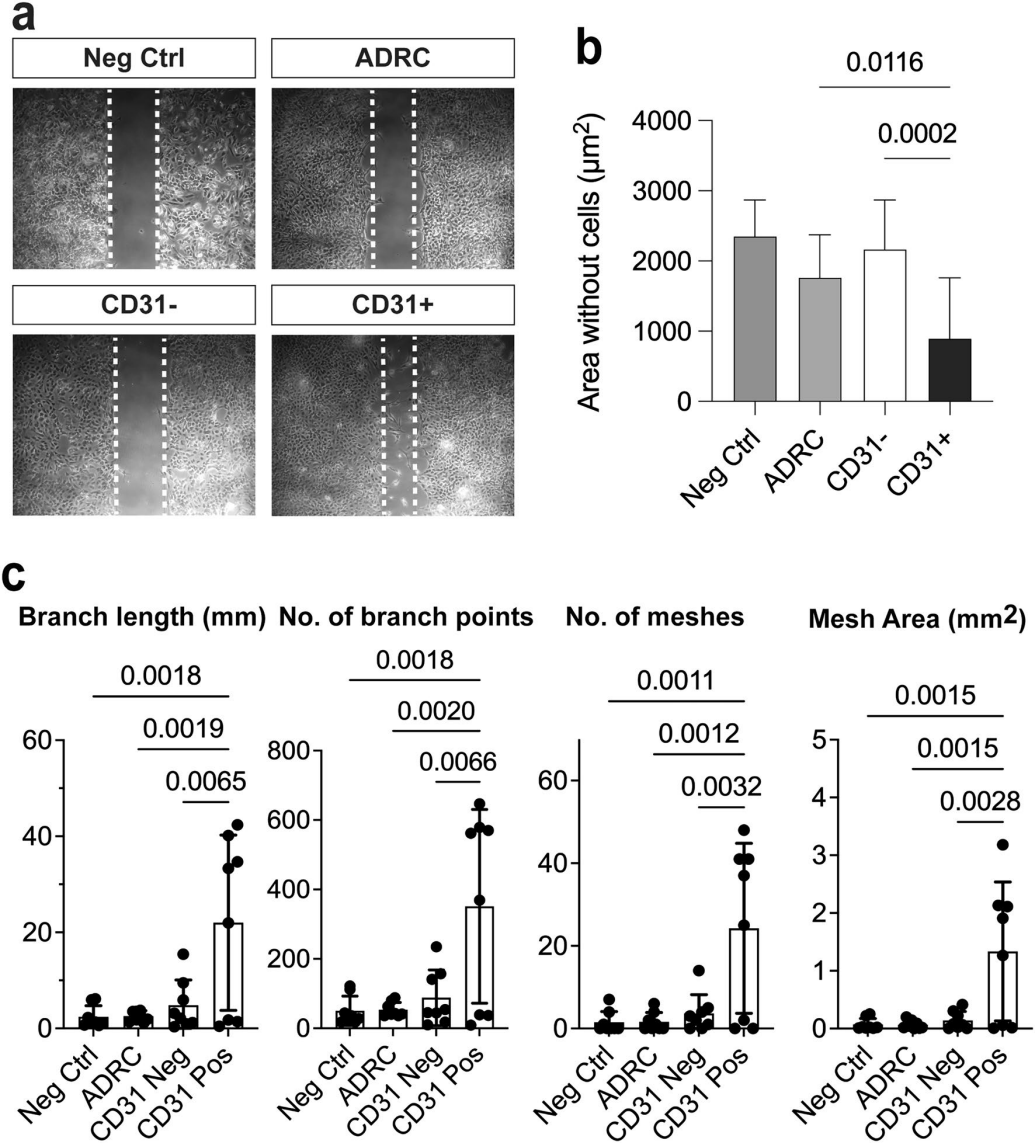
Confirmation of the superior paracrine effects of conditioned medium from CD31+  ADRCs. (**a**) Migration assay of primary mouse cavernosum pericytes (MCP)s after culture for 24 h in the MS-analyzed conditioned media obtained from ADRCs, CD31− and CD31+  ADRCs, respectively. Representative images for each condition are shown (Magnification × 2.5). The stippled lines indicate the boarder of the cell free area, representing a measure of the migration abilities of the MCPs. (**b**) MCP migration expressed as a function of the cell free area (µm^2^) following culture in CM from ADRCs, CD31− and CD31+  ADRCs, respectively. Quantification and statistical analyses were based on data from three experiments, using the MS-analyzed CMs obtained from three different donors. Each experiment consisted of 3 replicates for each tested cell type. Statistical analyses were performed using ordinary one-way ANOVA. Statistically significant p-values are shown in the figure panel. (**c**) Quantification and statistical analyses of the sprouting from mouse aortic ring explants after culture for 8 days in conditioned media from ADRCs, and CD31− and CD31+  ADRCs, respectively, evaluated based on total branch length (mm), number of branch points, number of meshes, and total mesh area (mm^2^). To overcome limitations in the amount of available MS-analyzed CM, equal volumes of the different CMs obtained from the three donors were pooled and used in one experiment with eight replicates for each CM-pool. Data are presented as means ± standard deviation (SD). Statistical analyses were performed using ordinary one-way ANOVA. Statistically significant p-values are shown in the figure panels.

We next tested the paracrine effect of the MS-analyzed CMs to eliminate possible interaction effects or feedback loops in the co-culture system. Since pericytes determine the location of sprout formation and guide endothelial cells^39^, we examined the migration of perivascular cells sprouting from CC explants using primary-derived mouse cavernous pericytes (MCP)s. The MCP identity was confirmed by NG2 and PDGFRβ expression (Supplementary Fig. 4b–c). Interestingly, CM from CD31+  ADRCs induced a significantly enhanced migration capacity as compared to CM from CD31− ADRCs and parent ADRCs (Fig. 5a,b). In the aortic ring assay, CM from CD31+  ADRCs was superior to CM from ADRCs, and the number of meshes and total mesh area in aortic rings cultured in CD31+  CM were greater compared to CD31− ADRC-derived CM (Fig. 5c). Thus, cultured CD31+  ADRCs secrete substances that induce angiogenesis.

### Single-cell RNA sequencing of CD31+  ADRCs reveals cell type heterogeneity and enriched angiogenic potential specifically in *PECAM1*^high^ expressing endothelial cells

**Figure 6:**
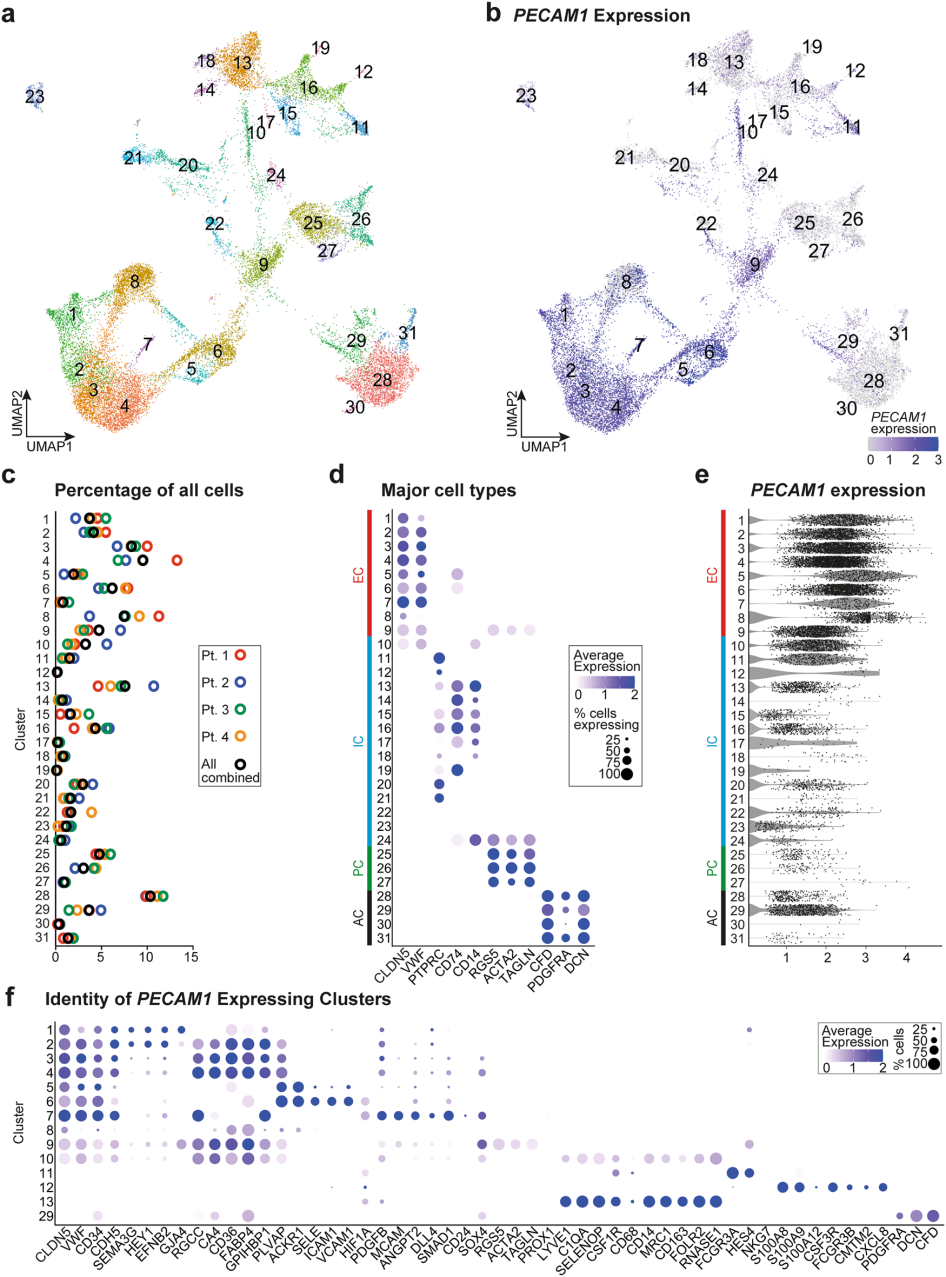
Single-cell RNA sequencing of 24,403 CD31+  ADRCs from 4 donors. (**a**) Uniform manifold approximation and projection (UMAP) 2D visualization of 24,403 CD31+  enriched ADRCs in 31 clusters. (**b**) Expression of the CD31-encoding *PECAM1*-gene in cells visualized in a 2D UMAP plot. (**c**) Percentage of total cells in each of the 31 clusters visualized for each of the four patient samples (Pt. 1–4) and for all four samples combined. (**d**) Dot plot showing marker-based assignment of the 31 clusters to four major groups: endothelial cells (EC)s (markers *CLDN5* and *VWF*), immune cells (IC)s (*PTPRC*, *CD74*, and *CD14*), perivascular mural cells (PC)s (*RGS5*, *ACTA2*, and *TAGLN*), and adipose stem and progenitor cells (AC)s (*CFD*, *PDGFRA*, and *DCN*). Color saturation of a dot indicates the average gene expression level in positive cells, while dot size reflects the percentage of cells in each cluster expressing the gene. (**e**) Violin plot of *PECAM1* expression levels in cells of the 31 clusters. (**f**) Dot plot of marker genes revealing the molecular identities of clusters 1–13 and 29, which were selected based on high average *PECAM1* expression and/or high percentages of *PECAM1* expressing cells.

To determine the cellular identity of the CD31+  ADRCs, we used high-resolution single-cell RNA sequencing (scRNA-seq) from samples obtained from 4 donors. The resulting quality-controlled, single-cell data included a total of 24,403 human CD31-selected ADRCs that were integrated and clustered using uniform manifold approximation and projection (UMAP) with 54 dimensions at resolution 1.0, resulting in identification of 31 cell clusters (Fig. 6a,b), all containing cells from each of the 4 samples (Fig. 6c). ADRCs expressing the CD31-encoding gene *PECAM1* were present in all 31 clusters (Fig. 6b,e), with 14 clusters (Clusters C1–C13, and C29) showing high average *PECAM1* expression and/or, high percentages of *PECAM1* expressing ADRCs (Fig. 6b,e). Based on canonical marker genes, the 31 clusters could be divided into four groups: (1) endothelial cells (EC)s (markers *CLDN5* and *VWF*), (2) immune cells (IC)s (markers *PTPRC*, *CD74*, and *CD14*), (3) perivascular mural cells (PC)s (markers *RGS5*, *ACTA2*, and *TAGLN*), and (4) adipose stem/progenitor cells (AC)s (markers *CFD*, *PDGFRA*, and *DCN*) (Fig. 6d).

We subsequently searched for cellular subsets with angiogenic potential. We identified the significantly upregulated genes in each of the 31 clusters, and secondly, clusters with over-representation of upregulated genes with the GO term: “GO:0001525 ~ angiogenesis”. We identified 14 clusters (EC clusters C1-C9, IC cluster C24, PC clusters C25 and C26, and AC clusters C28 and C31), representing 67.09% of all CD31+  selected cells, with over-representation of genes associated with the GO term: “GO:0001525 ~ angiogenesis”, suggesting that these clusters could play a role CD31+  ADRC angiogenic effect. As expected, the EC (and PC) clusters represent major contributors to the angiogenic signature, and noteworthy, a cluster of immature ECs (C7) contain the highest number of significantly upregulated angiogenesis-related genes (Supplementary Table 8). Similarly, the most stem cell-like ACs (C31), defined by expression of *DPP4*, *CD55*, and *SFRP4* (Supplementary Fig. 6b), possess a strong angiogenic profile. Finally, we observed a contribution from immune cell cluster C24, a subpopulation of monocytes where 98.0% express CD14. The majority (90%) of C24 cells also express the mural cell markers *RGS5*, *ACTA2*, and *TAGLN* (Supplementary Fig. 6a), suggesting this cluster defines a transitional stage between circulating monocytes and pericytes^45^.

**Figure 7:**
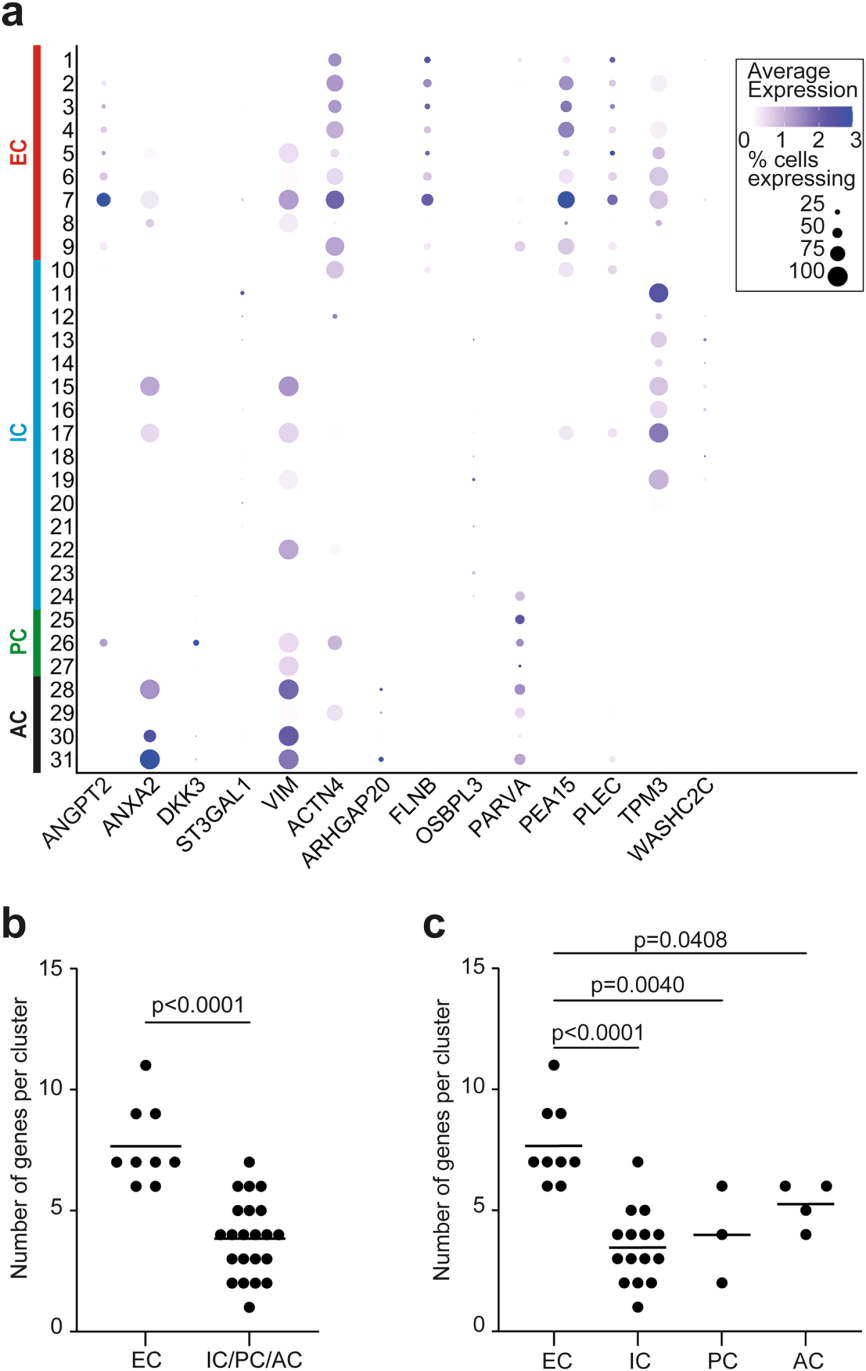
Several clusters of CD31+  selected ADRCs express genes encoding the 14 proteins that were significantly upregulated in the conditioned media from cultured CD31+  selected ADRCs. (**a**) Dot plot showing expression of the genes encoding the 14 proteins that were significantly upregulated in the conditioned media from cultured CD31+  selected ADRCs. Color saturation of a dot indicates the average gene expression level in positive cells, while dot size reflects the percentage of cells in each cluster expressing the gene. Note that particularly clusters 5 (presumptive postcapillary venule endothelial cells) and 7 (presumptive immature angiogenic endothelial cells) express high numbers of the genes, 9 and 11 genes, respectively, including the 4 genes encoding the secreted proteins ANGPT2, ANAX2, ST3GAL1 and VIM. (**b**) Number of genes, encoding proteins that were significantly upregulated in the conditioned media from cultured CD31+  selected ADRCs, expressed in EC clusters 1–9 compared to the remaining combined clusters 10–31 using the Mann–Whitney test. (**c**) Number of genes expressed in EC clusters 1–9 compared to the groups of IC clusters (10–24), PC clusters (25–27), and AC clusters (28–31), respectively using one-way ANOVA followed by the Dunnett test comparing every mean to the EC mean. The bars in panels b and c represent means. *AC* adipose stem and progenitor cells, *EC* endothelial cells, *IC* immune cells, *PC* perivascular mural cells.

We then investigated expression of genes encoding the 14 proteins that were significantly upregulated in the CM from CD31+  ADRCs (Fig. 4a). Indeed, all 31 clusters of CD31+  selected ADRCs contain cells expressing at least one of the 14 genes. However, clusters C5 (postcapillary venule ECs) and C7 (adipose-resident endothelial progenitor cells and immature angiogenic ECs, described in more details in the next section) expressed the highest numbers, 9 and 11 genes, respectively, including the 4 genes encoding the secreted proteins ANGPT2, ANAX2, and VIM (Fig. 7a). However, these clusters are relatively small, thus clusters 5 and 7 represent 1.96% and 0.80% of all CD31+  selected cells, respectively (Supplementary Table 6). Finally, the EC clusters C1-C9 contained significantly higher number of the 14 genes compared to the remaining combined clusters C10–C31 (p < 0.0001) and the IC, PC and AC clusters (Fig. 7b,c).

### *PECAM1*^high^ expressing endothelial ADRCs originate from blood vessels

Based on their high angiogenic potential, we looked closer into the origin of the EC cluster C1–C9 expressing PECAM1^high^. Overall, C1–C9 represent 46.93% of all CD31+  ADRCs, and they classify as endothelial cells containing the canonical endothelial markers *CLDN5*, *VWF*, and *PECAM1* (Fig. 6f). We searched for markers of endothelial cell types along the arterio-venous axis of the microvasculature. Cluster C1 (3.72% of CD31+  ADRCs) expresses markers of arteries and arterioles (*EFNB2*)^46^, *SEMA3G*, and *HEY1*^47^, as well as *CDH5* (VE-cadherin) and the gap junction gene *GJA4* (Fig. 6f). Cluster C2 (4.16% of all CD31+  ADRCs) expresses the other above-mentioned markers of arteriole except *GJA4*, and markers of capillary ECs (described below) indicating they originate from pre-capillary arterioles or post-arterial capillaries. Clusters C3, C4, and C9 (8.3%, 9.56%, and 4.74% of CD31+  ADRCs, respectively) appear to originate from capillary ECs expressing *RGCC*^48^, *CA4*^49^, *GPIHBP1*^50^, and the *CD36* and *FABP4* genes involved in fatty acid uptake, as well as *PLVAP* encoding endothelial cell-specific plasmalemma vesicle-associated protein that forms stomatal and fenestral diaphragms of capillaries and postcapillary venules, and regulates basal permeability, leukocyte migration and angiogenesis^51^. Interestingly, Cluster C9 also expresses markers of perivascular mural cells, namely *RGS5*, *ACTA2*, and *TAGLN* (Fig. 6f). Clusters C5 and C6 (1.96% and 6.2% of CD31+  ADRCs, respectively) express the HLA class II genes *CD74*, *HLA-DQB1*, *HLA-DRB1* (Supplementary Table 7), and have high expression of *PLVAP* together with *ACKR1*, which are both associated with postcapillary venules. Cluster C6 also expresses *SELE*, *ICAM1*, and *VCAM1* that encode proteins mediating rolling, firm adhesion, and trans-endothelial migration of leukocytes, suggesting that these cells may participate in immune modulation. Higher expression levels of *HIF1A*, *PDGFB*, *MCAM*, *ANGPT2*, and *DLL4* distinguishes cluster C7 cells (0.8% of all CD31+  ADRCs) that also express *SMAD1*, *CD24, SOX4* previously associated with immature ECs^52^ Fig. 6f). Notably, cluster C7 also has the highest *CD34* expression levels as well as expression of *ENG* (CD105), *MCAM* (CD156), and *CD200* (Supplementary Fig. 6a) associated with adipose-resident endothelial progenitor cells (AEPC)s^29^. Finally, Cluster C8 (7.48% of CD31+  ADRCs) expresses *CLDN5* and contains a subset of cells expressing *PECAM1* (36.5%) (Fig. 6e,f). This cluster contains upregulation of 66 ribosomal genes (Starting with “RPL*”, “RPS*” or “RSL*”), which may be the reason for the clustering of these cells (Supplementary Table 7). All non-endothelial clusters C10–C31 [including immune cells with high with average PECAM1 expression (C10–C13)] are further characterized in Supplementary Information S1 (Fig. 6d–f and Supplementary Fig. 6).

Thus, despite having a common therapeutic vascular phenotype, CD31+  ADRCs originate along the entire arterio-venous axis of the microvasculature, and therefore remain heterogenic in origin and likely also in function.

## Discussion

Herein, we identify and characterize a potent angiogenic subfraction within ADRCs, which may underlie their beneficial therapeutic effect in vascular repair.

Cell therapy for vascular regeneration include both autologous and allogenic cells obtained from various sources and with varying degrees of manipulation (i.e. selection, culturing) (reviewed in^23^). Mesenchymal stem cells (MSCs) derived either from adipose tissue (termed ASCs) or bone marrow are the most frequently used cell therapeutic products in general with 1000 + clinical MSC-trials registered world-wide^53^. Due to its accessibility, the adipose tissue stromal vascular fraction (referred to as ADRC herein) is a preferred cell source and since ex vivo expanded ASCs lack MHC class II expression, allogenic administration is largely without safety issues^54^. Despite being suitable as an off-the-shelf product, ASCs do not per se represent an improved product in comparison to ADRCs. Conventional culturing conditions profoundly impact cell phenotype and compared to the minimally processed heterogeneous ADRCs, the angiogenic properties of the more homogenous ASCs are reduced resulting in poorer outcomes in comparative in vivo studies^55^. This clearly suggests the existence of a more potent cell type (or synergistic mechanisms between multiple cell types) within the ADRCs. Thus, identification and characterization of such a cell type(s) will aid at realizing and maximizing the full clinical potential of ADRCs and -descendants for vascular regeneration in particular.

The pathogenesis of post prostatectomy erectile dysfunction (post RP-ED) is known to include a vascular component involving penile arterial insufficiency and/or veno-occlusive dysfunction and is thought to involve apoptosis of corporeal stromal cells, vascular smooth muscle, and endothelial cells. Using bone marrow-mononuclear cells (BM-MNCs), Yiou et al.^56^ observed significantly improved penile vascularization and normalized penile endothelial function in 8/11 post RP-ED patients in a dose-dependent manner. In agreement with preclinical ED studies^57,58^, Yiou et al. suggested the cellular therapy effect could be due to angiogenic repair. The BM-MNCs and ADRCs share many cell types, including mesenchymal stromal/stem cells and endothelial (progenitor) cells however, Yiou and co-workers did not address distributions of BM-MNC subpopulations in their study^56^. With this in mind, we re-visited our post RP-ED data from 15 urine continent men treated with ADRCs by a single intracavernous injection^2^. We found no significant relationship between total ADRC numbers and the patient-reported outcome measure IIEF-5, implying that the clinical effect could not be ascribed to the simple presence of a (critical) cell mass, but more likely to a specific subpopulation. Furthermore, the effect did not correlate with the number of CD34+ ADRCs (stem/progenitor cells among others), or CD73+/CD90+ ADRCs (mesenchymal cell populations), which is intriguing since these subpopulations represent the in situ ASCs. In contrast, the number of injected CD31+  ADRCs significantly correlated with the IIEF-5 score, implying an important role for CD31+  endothelial- and/or endothelial progenitor cells, likely due to their angiogenic/pro-angiogenic properties. In support of this notion, our data clearly demonstrate that enriched human CD31+  ADRCs exhibit superior paracrine angiogenic potential compared to both the CD31-depleted ADRCs and parent ADRCs. In the ex vivo angiogenesis assays, freshly isolated ADRC populations were seeded at the same density in insert wells and co-cultured with explants of murine corpora cavernosum or aortic ring explants. Analysis of *PECAM1* (encoding CD31) and *VWF* mRNA levels validated the CD31 enrichment strategy and showed sustained expression pattern in the insert cells until the endpoint (aortic ring assay day 8). Underscoring CD31+  ADRC potency, the total number of the actively transcribing insert CD31+  ADRCs were lower that of the corresponding ADRC and CD31− ADRCs due to higher growth rates of the two latter (data not shown).

The superior paracrine effect could be ascribed to components present in the CD31+  cells conditioned medium. Cell culture medium for quantitative proteomics was collected from single cultures of ADRC subsets to circumvent contribution from mouse explant tissue. As seen with the insert CD31+  ADRCs, *PECAM1* and *VWF* mRNA levels were sustained in the single cultures of CD31+  ADRCs up to day 8, whereafter levels were markedly reduced by day 15. This is in agreement with studies showing that CD31-expressing cells become overgrown by ASCs after 10 days of culturing^17,29^. Regardless, CM from day 15 CD31+  ADRCs outmatched the corresponding CD31− ADRC CM and ADRC CM when tested in pericyte migration assays as well as in the aortic ring assay. Furthermore, the CD31+  ADRC secretome showed enrichment of the pro-angiogenic factors ANGPT2^40,59^, DKK3^42^, VIM^60^, and ANAX2^61^. These 4 factors may contribute individually and synergistically to the observed effect. DKK3 has been shown to restore erectile function in diabetic mice by enhancing angiogenesis^62^. Due to the presence of abundant bovine serum proteins and inherent limitations of the relative quantification method (iTRAQ), other differentially expressed secreted proteins are likely not to have been detected which is also the case for proteins solely present in one type of CM. All 14 upregulated proteins were most frequently expressed in EC subtypes as evidenced by single-cell RNA sequencing of ~ 24,000 CD31+  enriched ADRCs. Our data suggest that the upregulation is not a culturing artefact but that ECs at the time of seeding are actively transcribing the relevant mRNA species. This underscores the importance of in-depth characterization of ECs, which are also the more *PECAM1* expressing cells in the enriched ADRC fraction. Notably, in the non-cultured CD31+  enriched ADRCs, *DKK3* mRNA was only identified in a pericyte subpopulation (C26), in agreement with other data suggesting that DKK3 is rarely expressed in normal endothelial cells^63^, but still critical for endothelial function and -regeneration^64^. Song et al.^62^, also suggested DKK3 to affect erectile dysfunction partially through pericytes and in general, pericytes play a vital role in angio-/vasculogenesis^65^. Whether DKK3 protein in the CM was produced by PCs expanding in vitro, or induced in ECs remains unanswered. However, the presence of various cell types in the CD31+  enriched fraction may have been advantageous for the angiogenic effect as several studies suggest synergistic regenerative effects from cell mixtures. Using a rat in vivo bone regeneration model, Sass an coworkers^66^ showed that CD31+  enriched cells from peripheral blood (PBMC) performed significantly better than further fractionated CD31+  CD14− or CD31+  CD14+ PBMCs. Revisiting ADRC (SVF) clinical data, Kilinic and co-workers^67^, reported improved efficacy using a 2:1 ratio of adipose-derived stromal/stem cells to adipose-tissue derived endothelial progenitor cells (EPC)s. Defined as CD34high CD45− CD31+  CD146 (MCAM)+^67^ these EPCs likely correspond to cells in our EC clusters, especially C7. C7 cells express the highest levels of ENG (CD105) and CD200 corresponding to recently reported AEPCs^29^.

At the single-cell level, the transcriptome and proteome vary considerably^68^ which could account for some of our CD31+  enriched ADRCs not expressing detectable *PECAM1* mRNA. It is also likely that contaminating CD31- ADRCs were co-purified due to non-specific aggregation or high-affinity interactions. Nevertheless, besides the EPC cluster C7, we characterized 7 distinct EC signatures expressing gradually overlapping markers for arterioles (C1, C2), capillaries (C3, C4, C9), and postcapillary venules (C5, C6). This is in line with scRNA-seq data from dermal ECs identifying 5 clusters (arterioles, capillary 1, capillary 2, postcapillary venules, venules)^69^, and 6 clusters (arterioles, post-arterial capillaries, pre-venular capillaries, postcapillary venules, venules, collecting venules)^70^.

In theory, cells with CD31/PECAM1 expression may have a homing advantage since CD31/PECAM1 engage in trans-homophilic interactions^71^. Interestingly, several non-endothelial clusters including cluster 10 and 11 also express high CD31 levels. These clusters are annotated as immune cells, suggesting CD31 plays a less uncharacterized role in these cells.

Collectively, our data support the notion that a potent angiogenic ADRC subpopulation exists, which may underlie the superior beneficial therapeutic effects as compared to cultured ASC counterparts. The CD31+  ADRC subset can be enriched and adjusted to meet GMP-compliance for direct autologous use or ex vivo expansion. In this regard the populations identified herein by scRNA-seq may serve as reference for future refinement of ADRC isolation- and expansion methodologies.

## Methods

All methods were carried out in accordance with relevant guidelines and regulations, and are reported in accordance with ARRIVE guidelines.

### Patient samples and data

Clinical outcome- and flow cytometry data used for the retrospective correlation analyses herein, were obtained in a previously described clinical phase 1 safety study for treating erectile dysfunction (ED)^2,3^. Clinical efficacy was addressed by evaluation of IIEF-5 and EHS scores, and the ADRC phenotype assessed by flow cytometry using markers for CD31, CD34, CD73 and CD90^3^.

Lipoaspirates for ADRC isolation (n = 10) were obtained from placebo patients enrolled in two separate phase 2 RCTs using ADRCs for cell therapy of ED (unpublished; awaiting final data analyses) and lymphedema (submitted). All patients gave written informed consent before participation.

### Regulatory approvals

The ED-trials followed ATMP guidelines and were approved by The Danish Health and Medicines Authority [EUdra-CT nos. 2013–004220–11 (Phase 1) and 2015–005140-33 (Phase 2)], the Danish National Ethics Committee [nos. 37054 (Phase 1) and 51,658 (Phase 2)] as well as the Danish Data Protection Agency [nos. 2008–58–0035 (Phase 1 and 16/2816 (Phase 2)]. The Phase 1 ED study was registered at ClinicalTrials.gov (NCT02240823). All studies were performed in accordance with the Declaration of Helsinki and ICH-GCP guidelines.

### Animals

8-week-old C57BL6 mice (male and female) were obtained from Janvier Labs (Le Genest-Saint-Isle, France), and kept in an animal facility, with controlled temperatures, a 12-h light/dark cycle. For collection of penile and aortic tissue, animals were sacrificed with CO_2_. All experimental protocols were approved by The Regional Committees on Health Research Ethics for Southern Denmark.

### Isolation of adipose derived regenerative cells (ADRC)

ADRCs were isolated from human lipoaspirates as described in detail previously^3,72^.

### Magnetic cell sorting enrichment of CD31+  ADRCs

Enrichment of CD31 positive and -negative ADRC subpopulations was accomplished by magnetic cell sorting (MACS) using MS separation columns (Miltenyi Biotech) under RNase-free conditions. Initially, the isolated ADRC was subjected to red cell lysis (RBC buffer, Miltenyi Biotech, cat no.130-094-183). Magnetic labelling was performed using a CD31 microbead kit (Miltenyi Biotech) after which cells were sorted on an OctoMACS™ Separator (Miltenyi Biotech). Hereby, CD31+  and CD31− ADRCs were obtained based on a positive or negative selection for the CD31 marker.

### Flow cytometry

CD31-enrichment and -depletion of ADRCs was verified by flow cytometry using an anti-CD31 antibody (CD31-Vioblue antibody, Miltenyi Biotech cat. no. 130-106-503). Sample acquisition was performed on a BD™ LSRII flow cytometer and analysed using the FACSDiva™ software v8.0.1 and FlowJo v10.

ADRC flow cytometry data from the phase 1 ED clinical trial employed herein, were originally analysed using antibodies against CD34 (PECF594, clone 581), CD31 (Alexa Fluor® 647, clone WM59), CD73 (APC, clone AD2), CD90 (APC, clone 5E10), and appropriate isotype controls.

### Conditioned media

ADRCs, CD31+  and CD31− ADRCs were cultured in endothelial cell basal medium (PromoCell, cat no. C-22210) with 1% penicillin/streptomycin (PS) (= EBM) supplemented with 2.5% fetal bovine serum (FBS) for 15 days without media exchange. Conditioned media (CM) was centrifuged at 2500×*g* for 15 min. After media collection, RNA was isolated from the ADRC, CD31+  and CD31− ADRCs as described below.

### Corpus cavernosum explant co-culture assay

Mouse corpus cavernosum was prepared according to Ghatak et al.^73^. Briefly, following termination, the penile tissue from 6 to 8-week-old C57BL6 mice (Janvier, France) was dissected. For the ex vivo assay, the tissue was cut into three/four 1 mm pieces, and the explants were plated on growth factor reduced Matrigel (Corning, cat no. 356231). Following polymerization, explants were supplemented with 1 ml EBM and 2.5% FBS and incubated at 37 °C with 5% CO_2_^48^.

After 24 h, media was replaced with 1.5 ml fresh EBM with 2.5% FBS. Next, Nunc™ polycarbonate Cell Culture Inserts with 0.4 µm pore size (Thermo Scientific catalog no.140620) were placed above the explant, and 20,000 cells (ADRCs, CD31− or CD31+) were seeded in 500 µl EBM with 2.5% FBS (3 wells pr. cell type). The plate was then incubated at 37 °C with 5% CO_2_ for 15 days without medium exchange.

Structurally distinct regions of tubular sprouting from corpus cavernosum explants were comparable to the regions previously described for the aortic ring assay^37^. The tubular network was quantified in defined, fixed areas (see Supplementary Fig. 2c) using the ImageJ software (Fiji) with an angiogenesis plugin. The angiogenesis evaluation was based on number of nodes, total length of tubes, number of meshes/mm^2^ and mesh coverage.

### Aortic ring ex vivo assay

The aortic ring assay was set up as previously described^74^. Briefly, the thoracic aorta was dissected from 8 to 12 weeks old C57BL6 mice into 0.5–0.7 mm wide rings, and serum-starved in EBM for 24 h. For indirect co-culture assay, one aortic ring per well of a 24-well Nunc™ Carrier Plate was embedded between two 40 µl drops of Matrigel with 700 µl EBM containing 2.5% FBS. Next, Nunc™ polycarbonate Cell Culture Inserts with 0.4 µm pore size were placed above the aortic rings and 50,000 cells per insert (ADRCs, CD31+  or CD31− ADRCs) were seeded in 500 µl EBM with 2.5% FBS (8 wells pr. cell type). To test CM effects, Matrigel embedded aortic rings were cultured in 500 µl CM from ADRC’s, CD31+  or CD31− ADRCs. The plate was cultured at 37 °C for 8 days, at which time, pictures were acquired with phase contrast microscopy (5 × magnification) and analyzed using the angiogenesis plug-in in ImageJ.

RNA from the ADRCs, CD31− and CD31+  ADRCs cultured in the inserts was collected using Tri Reagent® (ThermoFisher Scientific, cat no. AM9738).

### Stable isotope labeling of protein samples with TMT-10 plex

Proteins from CM were isolated by transferring the supernatant to five equivalents of ice-cold acetone. Proteins were reduced using 5 mM dithiothreitol (DTT), followed by 15 mM iodoacetamide blocking before trypsination overnight at 37 °C at a protein:trypsin (Promega, Madison, WI, USA) ratio of 50:1 w/w. 10 µg of the tryptic digest was labeled with a 10-plex TMT-kit (Thermo Scientific), resuspended in anhydrous ethanol, and a 40 µg sample was labeled according to the scheme in Supplementary Table 1. Labeled samples were pooled in equal ratios, dried in a vacuum centrifuge, re-dissolved in 50 µl trifluoroacetic acid solution (0.1%), purified, loaded on a reverse phase microcolumn (equal w/w amounts of Poros R2 and Oligo R3 material) and fractionated by high pH liquid chromatography as described^75^.

The Fractions were analyzed by RP‐nanoLC‐MS/MS on an Orbitrap Eclipse mass spectrometer (Thermo Fisher Scientific) equipped with a nano HPLC interface (Dionex UltiMate 3000 nano HPLC) as described^75^. Raw data files were quantified using Proteome Discoverer version 2.4 (Thermo Scientific) as previously described using human and bovine database searches^76^.

### Isolation of primary murine cavernous pericytes

Penile tissue from mice was prepared and embedded in Matrigel as described for the Corpus Cavernosum explant assay. The Matrigel drop and the tissue was plated in a 60 mm petri-dish. After Matrigel polymerization, the petridish was supplemented with EBM containing 20% FBS. The medium was changed every 4 days. Cells sprouted from the corpus cavernosum explants (4 per dish), and after approximately 2 weeks, they became confluent. Only cells migrating out of the Matrigel onto the plastic surface of the petri-dish, were further sub-cultivated. These migrated pericyte cells were trypsinized and seeded at 10,000 cells/cm^2^ density in EBM with 20% FBS for further experiments.

### Migration in vitro assays (with conditioned media)

To test the angiogenic effect of conditioned medium from CD31+  ADRCs, we adopted the wound healing assay using pericyte migration. Primary pericytes were seeded in culture inserts (ibidi culture-insert 2 well, ibidi GmbH, Martinsried, Germany) at a density of 25,000 cells per well. After allowing cells to attach overnight, we removed the culture inserts creating a cell-free gap and washed the cells with sterile PBS to remove non-adherent cells. We then provided 300 μl of CM from ADRC, CD31+  , or CD31− ADRCs, or EBM with 2.5% FBS as a control. Images of cell-free gaps were taken immediately after removing inserts with a bright field microscope at 5 × magnification. We monitored the gap for 24 h after culturing cells in respective CM, at which point images of the gaps were captured again. The cells migration ability was evaluated by the area of the gap they had covered in 24 h using ImageJ.

### Immunofluorescence staining

Cultured pericytes and pericytes sprouting from corpus cavernous explant in Matrigel, were fixed with 4% neutral buffered formalin (NBF) for 15 min at room temperature and permeabilized with 0.1% Triton X-100/0.1% Na-Citrate/PBS for 10 min on ice. Cells were blocked with 2% BSA in PBS and stained with rabbit anti-NG2 (Millipore #AB5320, 1:200), mouse anti-PDGFRß (Novus biologicals#NBP1-43349, 1:200), rabbit anti-αSMA (abcam#GR195159, 1:100) or mouse anti-CD31 (Novus Biologicals#NB600-562, 1:200) at 4 °C overnight. Following a 90 min. incubation with donkey secondary antibodies (Alexa 488 or Alexa 555 labelled, Invitrogen. 1:200), the nuclei were stained with DAPI (VWR, cat no. 172867). Finally, images were captured at a 20 × magnification using a Leica DMI4000B instrument with a Leica DFC340 FX Digital Camera.

### RNA purification and RT-qPCR

RNA was extracted from samples of ADRCs, CD31+  and CD31− ADRCs, which were obtained from the aortic ring co-culture assay, the CM production set-up, and from the in vivo Matrigel cell culture. The samples were homogenized, and extraction of the total RNA was performed using the Tri Reagent® protocol (ThermoFisher Scientific). The RNA quantity and purity was assessed by nanodrop measurements (Nanodrop® Technologies). The mRNA was reversed transcribed using a High-Capacity cDNA kit (Applied Biosystems, Thermo Fisher Scientific) and the RT-qPCR reaction was performed using Power SYBR® Green PCR kit (Applied Biosystems). Primers specific for the following target genes were used: *PECAM1* (encoding CD31), *DKK3* (Dickkopf 3), *ANGPT2* (Angiopoietin 2), *ANXA2* (Annexin 2) and *VIM* (Vimentin) (Integrated DNA technologies, USA) (Supplementary Table 2). The RT-qPCR analysis was performed on a QuantStudio 7 instrument (Applied Biosystems). The data from the RT-qPCR was analyzed using the qBase + software (Biogazelle, Belgium) and normalized against multiple housekeeping genes, chosen based on the geNorm analysis in qBase+.

### Single-cell RNA sequencing

To enable single-cell RNA sequencing (scRNA-seq), at least 10^6^ CD31+  ADRCs from each of four subjects (one male and three females) were methanol-fixed and stored at – 80 °C essentially as previously described^77^. Briefly, the cells were resuspended in 500 µl PBS with 1% BSA and 1 U/µl RNAsin PLUS RNase Inhibitor (Promega, Cat.no. N2615) and passed through a 40 µm Flowmi® Cell strainer (VWR, cat.no. 734-5950) to create a single-cell suspension. The cells were fixed for 30 min at – 20 °C and subsequently stored at – 80 °C until use.

Before sequencing, fixed cells were thawed and rehydrated (3 × saline sodium citrate, 0.04% BSA, 1 U/µl RNAsin Plus, 40 mM Dithiothreitol). Immediately following rehydration, 8000 cells were loaded onto the 10 × Genomics Chromium controller (10 × Genomics, PN110203). Libraries were prepared according to the instructions of the manufacturer using the 10 × Genomics Single-Cell 3′ v3, Chromium Single Cell B Chip Kit, 48 runs (10 × Genomics, 10 × Genomics, PN-1000073) and sequenced on an Illumina NovaSeq 6000 System (10 × Genomics, 20012850). Cell samples were kept for RNA purification, and RIN-values were measured using RNA 6000 Nano kit (Agilent, cat no. 5067-1512). The RIN-values were 7.25, 6.80, 5.00, and 7.53 for the four single-cell samples Pt. 1–4, respectively.

### scRNA-seq data analysis

Using Cell Ranger software (v. 3.1.0), Illumina raw sequencing output files were demultiplexed and aligned to the GRCh38 human reference genome and human transcriptome reference (ENSEMBL version 102) to create a single-cell feature count matrix for each sample. The output metrics generated by Cell Ranger are listed in Supplementary Table 5. Overall, the four patient samples (Pt. 1–4) had an estimated total cell number of 25,928 and were sequenced with a coverage of around 220 million total reads per sample (250,368,339, 210,296,540, 223,244,205, and 195,915,808 for Pt. 1–4, respectively) corresponding to a mean of around 34,000 reads per cell (specifically 30,663, 21,555, 49,205, and 56,459 for Pt. 1–4, respectively) (Supplementary Table 5). All subsequent analyses were performed using R Version 4.1.0 and the *Seurat* R software package (Version 4.0.2). First, the data was converted to single-cell *Seurat* objects retaining genes expressed in at least five cells, each dataset was filtered, excluding low-quality cells with mitochondrial RNA contents (percent.mt) over 10%, or number of genes detected per cell (nFeature_RNA) below 200. We also excluded cells with nFeature_RNA above an upper threshold, which was set individually for each of the four samples: 4600, 4700, 4650, and 4450 for Pt. 1–4, respectively. A total of 24,403 cells passed these quality filtering steps. Next, each dataset was subjected to normalization with a scale factor of 10,000 using the “LogNormalize” method, identification of the top 2000 most variable genes using the “FindVariableFeatures”-function, and scaling with the following variables regressed: "nFeature_RNA" and "percent.mt". Next, the 4 datasets were integrated using the “FindIntegrationAnchors” and “IntegrateData” commands in *Seurat*^78^. The integrated dataset was scaled, and principal components were identified. After doing a Jackstraw plot, in which a p-value is assigned to each principal component, uniform manifold approximation and projection (UMAP) was run and cell “neighbors” were identified (both with the reduction dimensions set to 54), and finally cells were clustered and visualized with the resolution parameter set to 1.

Differential gene expression analysis was performed for each individual cluster using the “FindMarkers”-function in *Seurat*, which compares the gene expression levels in a specific cluster with the corresponding genes in the cells of all other clusters using a Wilcoxon Rank Sum test with an adjusted p-value cutoff of 0.05 based on Bonferroni correction. All significantly differentially expressed genes are listed in Supplementary Table 7. Subsequently, the differentially expressed genes for each cluster were analyzed for significantly enriched gene ontology (GO) terms using Database for Annotation, Visualization, and Integrated Discovery (DAVID version 6.8; https://david.ncifcrf.gov/). All significantly enriched GO terms are listed in Supplementary Table 8.

### Statistics

Quantitative data are expressed as mean ± standard deviation (SD) and statistical analyses were performed using the GraphPad Prism software version 9. Statistical significance was set at *p* < 0.05. and in each case determined using the appropriate statistical method following normality testing, as denoted in the corresponding figure legend.

### Supplementary Information

Supplementary Figures.

Supplementary Tables.

## Data Availability

All scRNA-seq data have been deposited in the ArrayExpress database at EMBL-EBI with accession code: E-MTAB-12865 (link: https://www.ebi.ac.uk/biostudies/arrayexpress/studies/E-MTAB-12865?key=3cfd12ed-7e2d-4b87-89fe-45f508f547ac. Sensitive genetic information was eliminated from RNA-seq raw files applying BAMboozle^79^ prior to depositing. The mass spectrometry proteomics data have been deposited to the ProteomeXchange Consortium via the PRIDE partner repository with the dataset identifier PXD040388. The rest of the data underlying this article are available from the corresponding author (soeren.sheikh@rsyd.dk) or co-last author (charken@health.sdu.dk) upon reasonable request.
