# Supplementary material for: CD31 defines a subpopulation of human adipose-derived regenerative cells with potent angiogenic effects
Source: Sci Rep. 2023 Sep 1;13:14401. doi: 10.1038/s41598-023-41535-1 (PMC10474028; doi:10.1038/s41598-023-41535-1)
Supplement: Supplementary file 1 — Supplementary Figures. [file 41598_2023_41535_MOESM1_ESM.pdf]

CD31 defines a subpopulation of human adipose-derived regenerative cells with potent angiogenic effects.

Supplementary Information

Pratibha Dhumale[1,2§] and Jakob Vennike Nielsen[2§], Anne Cathrine Schmidt Hansen[2], Mark Burton[3], Hans Christian Beck[1,2], Mads Gustaf Jørgensen[1,5], Navid Mohamadpour Toyserkani[4], Martha Kirstine Haahr[5], Sabrina Toft Hansen[1,5], Lars Lund[1,5], Mads Thomassen[1,3], Jens Ahm Sørensen[1,4], Ditte Caroline Andersen[1,2], Charlotte Harken Jensen[1,2$] and Søren Paludan Sheikh[1,2$*]

[1]Department of Clinical Research, University of Southern Denmark (SDU), Odense, Denmark.

[2]Department of Clinical Biochemistry, Odense University Hospital (OUH), Denmark.

[3]Department of Clinical Genetics, OUH, Denmark.

[4]Department of Plastic Surgery, OUH, Denmark. Research Unit for Plastic Surgery, Department of Clinical Research, SDU

[5]Department of Urology, OUH, Denmark.

[§]PD and JVN contributed equally to the study. [$]CHJ and SPS are joint senior-authors.

Correspondence to: Søren P Sheikh*, e-mail: soeren.sheikh@rsyd.dk

Contents:

Supplementary Figure 1

Supplementary Figure 2

Supplementary Figure 3

Supplementary Figure 4

Supplementary Figure 5

Supplementary Figure 6

List of Supplementary Tables

Supplementary information S1: Characterization of non-endothelial single-cell RNA sequencing clusters C10-C31.

Supplementary References

Supplementary Figure 1.

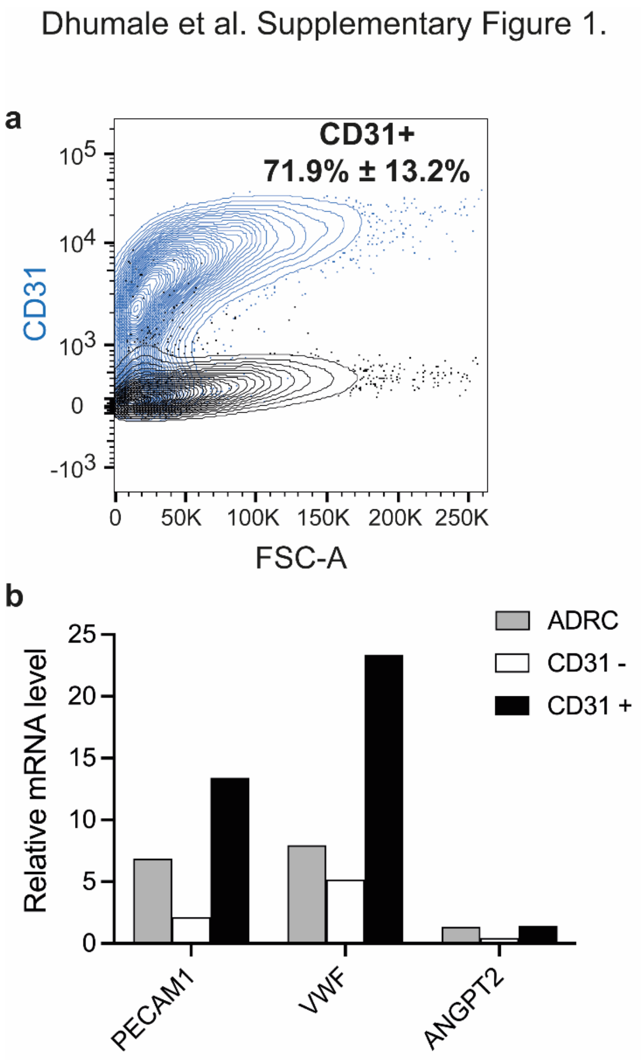

Supplementary Figure 1.

a. Representative flow cytometry plot of CD31+ selected ADRCs showing that 71.9% ± 13.2% of the CD31+ selected ADRCs (n=3) express the CD31 surface marker. The bivariate contour plot of recorded, gated events depicts the intensity of CD31-Vioblue antibody staining as a function of their forward scatter (FSC, indicating size).

b. Relative mRNA expression of *PECAM1*, *VWF*, and *ANGPT2* in freshly isolated ADRC, and the CD31+ and CD31- ADRC subsets, respectively, all normalized to four stably expressed housekeeping reference genes (*B2M*, *TBP*, *PGK1* and *HPRT1*).

Supplementary Figure 2.

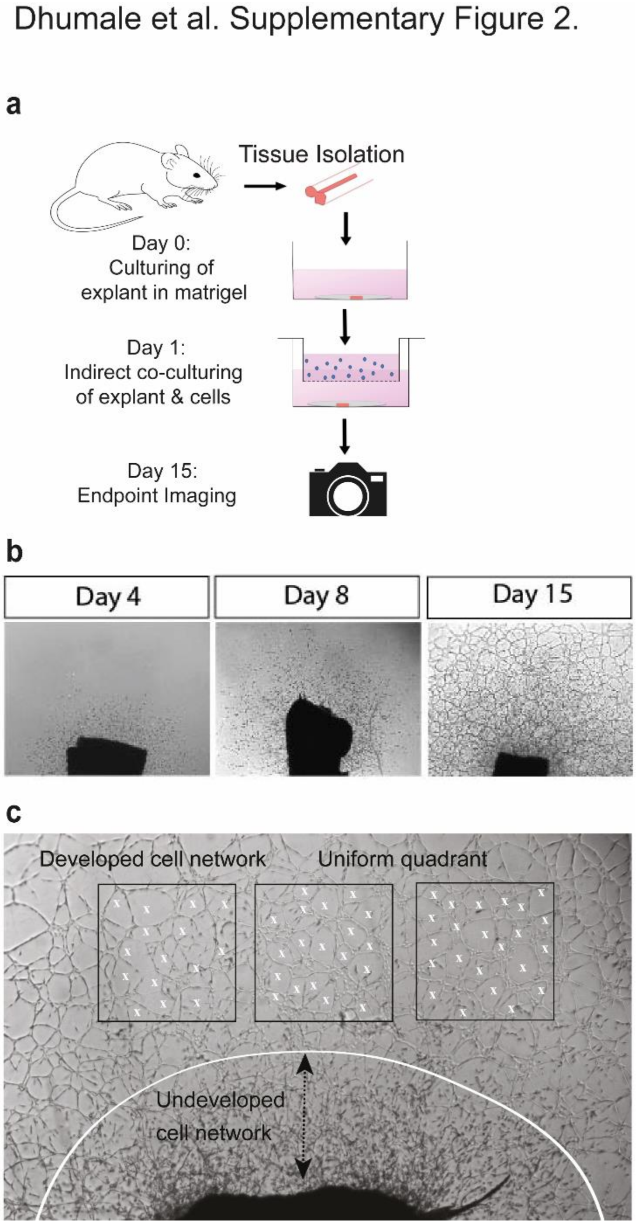

Supplementary Figure 2.

a. Schematic representation of the workflow of the corpus cavernosum explant co-culture assays.

b. Representative images of time-dependent sprouting from mouse corpus cavernous explants grown in EGM2 angiogenesis medium (initial setup using defined, optimal medium) for 4, 8, and 15 days, respectively.

c. Sprouting networks from corpus cavernous (CC) explants are divided into two concentric regions based on structural differences: 1) an unstructured area characterized by high cell numbers but low structural organization in proximity to the CC-explant, and 2) a well-developed area characterized with higher structural organization of tubes and establishment of mesh-like structures, located more distal to the CC-explant. The border between these two areas is indicated by a solid white line. Three uniform quadrants, in which the quantification of angiogenic parameters were performed, are indicated by black-lined open boxes.

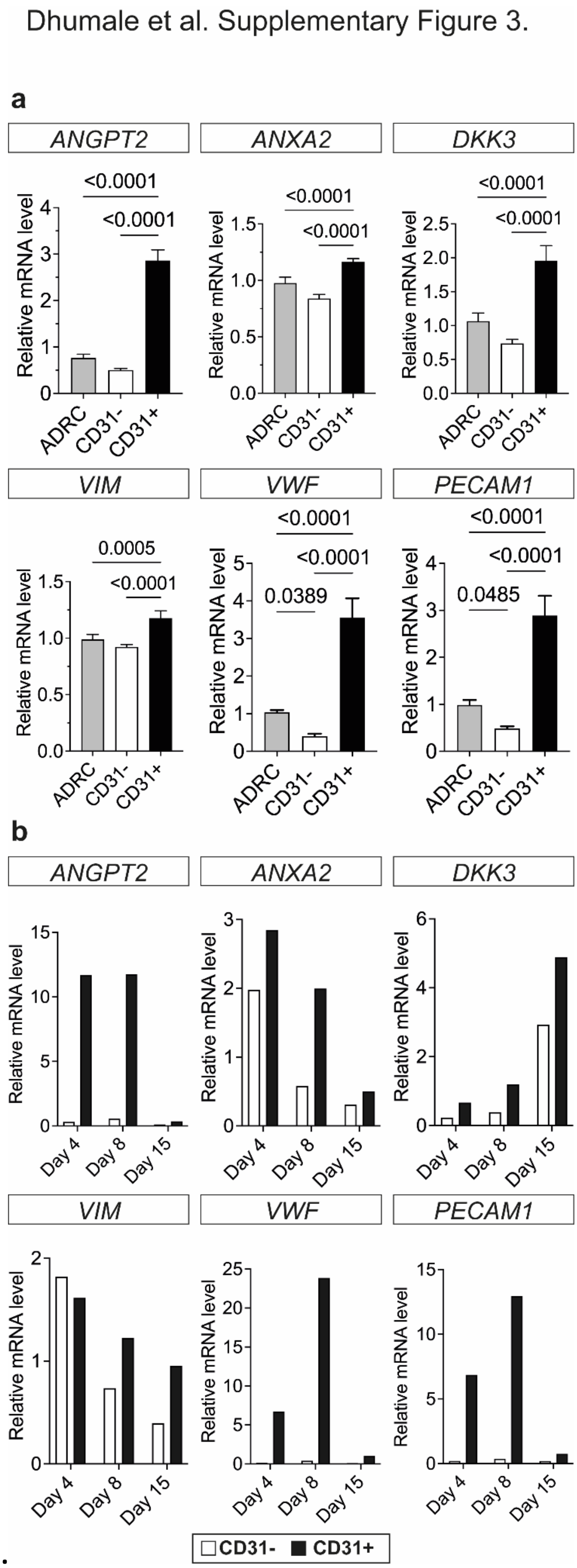

Supplementary Figure 3.

Supplementary Figure 3.

a. Relative mRNA levels of *ANGPT2, ANXA2, DKK3, VIM*, *VWF*, and *PECAM1* in ADRC, and CD31- and CD31+ ADRCs after 8 days of co-culture with mouse aortic ring explants.

The data was based on cells from one donor, and eight replicates for each of the three cell populations. To obtain sufficient material, two replicates were pooled in relation to RNA extraction and RT-qPCR performed (in technical triplicates) on the resulting 4 replicates per population. The mRNA expression was normalized to the expression of the reference genes *B2M* and *TBP*, based on the geNorm analysis performed in qBase+ (CV = 0.066, M = 0.191). Statistical analyses were performed using ordinary one-way ANOVA. Statistically significant p-values are shown in the figure panel.

b. Relative mRNA levels of *ANGPT2, ANXA2, DKK3, VIM*, *VWF*, and *PECAM1* in CD31- and CD31+ ADRC subsets cultured for 4, 8 and 15 days, respectively. The time course RT-qPCR data was based on cells from one donor, with 3 technical replicates for each time point. The mRNA expression levels were normalized to the expression of the housekeeping genes *GAPDH* and *PGK1*, based on the geNorm analysis performed in qBase+ (biogazelle) (geNorm CV = 0.151 and geNorm M = 0.437). Data are presented as means of the 3 technical replicates.

Supplementary Figure 4.

Dhumale et al. Supplementary Figure 4.

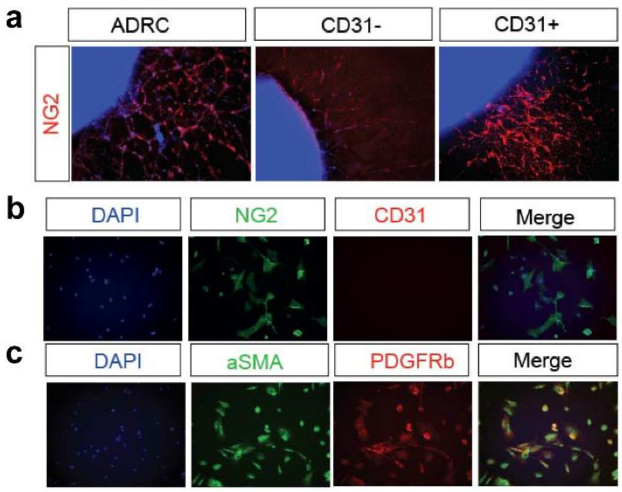

Supplementary Figure 4.

a. Sprouting cells from corpus cavernosum explant assays stain positively with the perivascular marker NG2; but not with the endothelial marker CD31, suggesting the presence of pericytes in the capillary-like structures.

b. primary mouse pericytes (MCP) isolated from corpus cavernosum explants were stained with perivascular and endothelial markers. The top row shows that the cells co-stained for NG2 and DAPI, but not for CD31, making them non-endothelial and instead perivascular e.g., pericytes. This is further confirmed in the bottom row by positive co-staining of DAPI, αSMA and PDGFRβ. Images were captured at 20x magnification and the scale bar 20 μM.

Supplementary Figure 5.

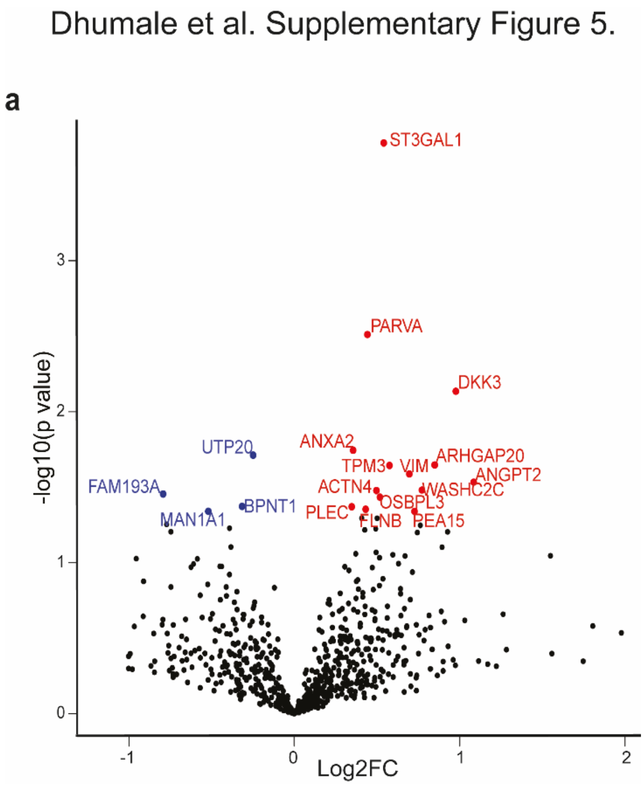

Supplementary Figure 5.

Volcano plot representation of the differentially expressed proteins in the CD31+ ADRC vs CD31- ADRC conditioned media. The 14 significantly upregulated and 4 significantly downregulated proteins in the CD31+ ADRC vs CD31- ADRC conditioned media, as identified by individual t-tests with a p-value of 0.05, are indicated by red and blue colors, respectively.

Supplementary Figure 6.

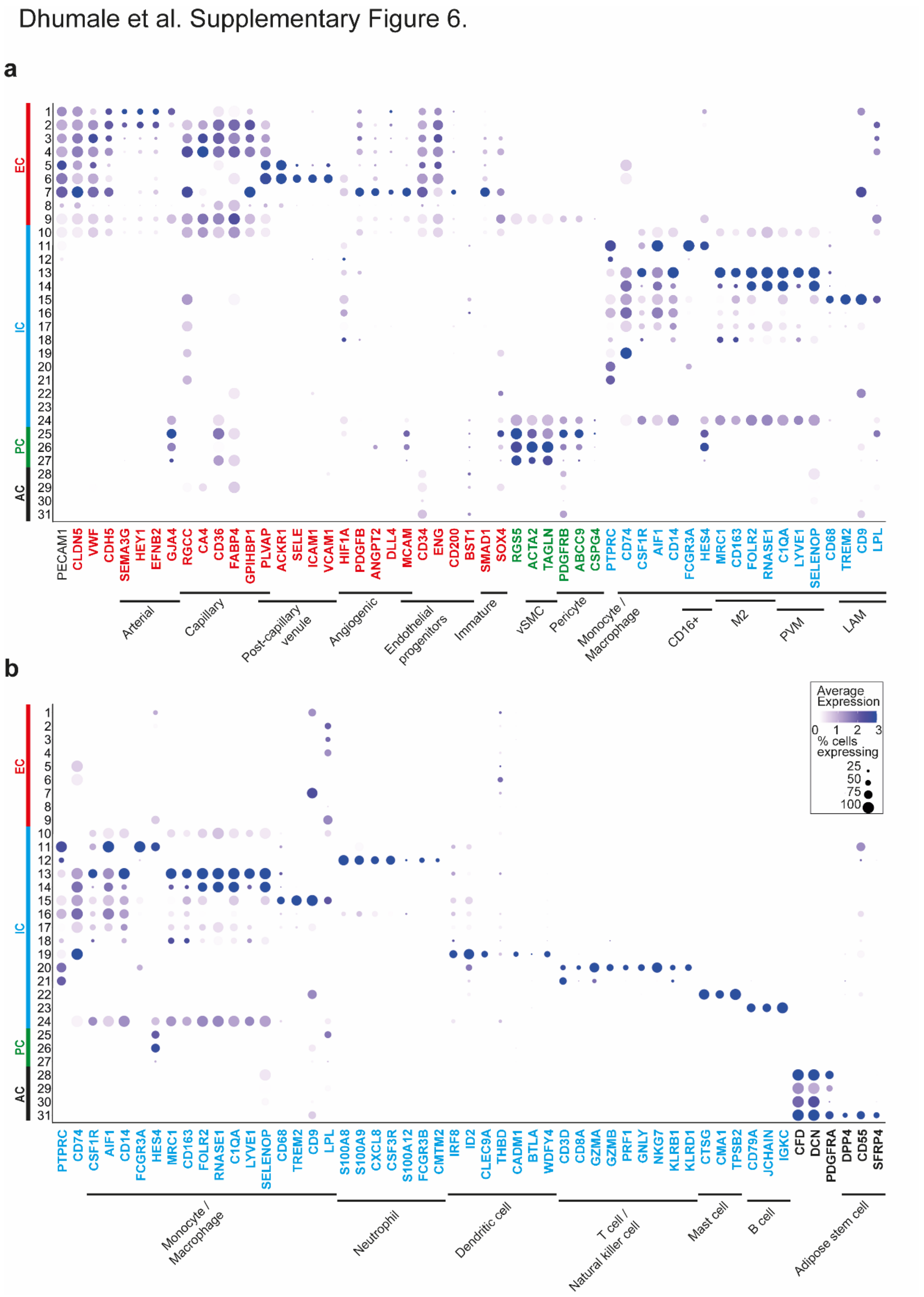

Supplementary Figure 6. Dot plot of all marker genes used to establish the molecular identities of the 31 CD31+ ADRC **clusters.**

Marker genes used to establish the identities of the endothelial cell (EC) subtypes (clusters 1-9) are depicted in bold red. Marker genes of immune cell (IC) subtypes are in bold blue (clusters 10-24), marker genes of perivascular mural cell (PC) subtypes are in bold green (clusters 25-27), and marker genes of adipose stem and progenitor cells (AC) are in bold black (clusters 28-31).

Color saturation of a dot indicates the average gene expression level in positive cells, while dot size reflects the percentage of cells in each cluster expressing the gene.

Abbreviations: AC: adipose stem and progenitor cells; EC: endothelial cells; IC: Immune cells; PC: perivascular mural cells; vSMC: Markers of vascular smooth muscle cells; CD16+: Markers of CD14-/CD16+ non-classic monocytes; M2: Markers of M2-polarization of monocytes and/or macrophages; PVM: Markers of Perivascular Macrophages [83]; LAM: Markers of Lipid Associated Macrophages [83].

List of Supplementary Tables

Supplementary Table 1. Scheme of Stable Isotope Labeling of Protein Samples for RP-nanoLC-MS/MS analysis.

Supplementary Table 2. Primer sequences used in this study. Forward and reverse primers for all target genes and reference genes can be seen, as well as the ID on the one TaqMan assay used for the RT-qPCR performed on the *in vitro* cultured cells.

Supplementary Table 3. List of 997 proteins detected in conditioned media from ADRCs, CD31+ or CD31- ADRCs after 15 days of culture, N=3. A total of 680 quantifiable proteins are highlighted with a light-blue background.

Supplementary Table 4. Differentially expressed Proteins in CM of CD31+ and CD31- ADRCs, respectively, and their cellular functional categories and GO terms associated with them are listed.

Supplementary Table 5. Single-cell RNA sequencing CellRanger Output Metrics.

Supplementary Table 6. Single-cell RNA sequencing data. Cell numbers of the 4 donor samples and distribution of cells in 31 clusters.

Supplementary Table 7. Single-cell RNA sequencing data. Cluster marker genes for each of the 31 clusters generated using the “FindMarkers”-function in Seurat.

Supplementary Table 8. Single-cell RNA sequencing data. Gene Ontology Analyses of Significantly upregulated Cluster Marker Genes for each of the 31 clusters.

Supplementary information S1:

Characterization of non-endothelial single-cell RNA sequencing clusters C10-C31.

Fifteen immune cell clusters (clusters C10-C24), representing 28.58% of all CD31+ ADRCs, were identified based on their expression of the canonical hematopoietic marker *PTPRC* (encoding CD45), and/or *CD74* (HLA class II), and/or *CD14* (Figures 6d-f), or additional markers of myeloid and lymphoid lineage (Supplementary Figure 6a-b). Four of these clusters (C10-C13) contained subsets of cells with high average expression of *PECAM1* or contained high percentages of cells expressing *PECAM1* at lower levels. Cluster C13 (7.59% of all CD31+ cells) has a larger subset of cells expressing *PECAM1* at low levels (Figure 6b-f). Cells from this cluster further express *CSF1R* a marker of mononuclear phagocytes [1], *AIF1* a marker of monocyte/macrophage lineage and dendritic cells subsets [2], and high levels of *CD14* together with markers associated with M2-polarization: *MRC1*, *CD163*, *FOLR2*, *RNASE1*, and genes expressed by perivascular macrophages: *LYVE1*, *C1QA*, *SELENOP* [3], indicating that these cells are classical CD14+ monocytes/macrophages. Cluster C10 (3.28% of all CD31+ cells) also has many cells expressing *CD14* (84.8%) and *CSF1R* (60.4%), as well as the above-mentioned markers of M2-polarization and of perivascular macrophages. Although these genes are expressed at lower levels, the cluster appears to have some similarities with cluster C13 (Figure 6f). Interestingly, C10 also expresses the EC markers *CLDN5*, *VWF*, *CD34*, and *CDH5* together with capillary marker genes, all at relatively low levels (Figure 6f). Given that 62.1% of the cells express *LYVE1*, which is also expressed by lymphatic ECs (LEC)s [4], we checked for expression of an additional pan-LEC marker *PROX1*, which is not expressed in cluster 10, ruling out that these cells are LECs (Figure 6f).

PECAM1 is expressed at moderate levels in cluster C11 (1.53% of all CD31+ cells) that further express *CSF1R* indicating a phagocyte identity. Since C11 has no *CD14*-expression but expresses

FCGR3A (CD16A) and *HES4*, this implies that these cells are non-classical CD16-positive monocytes as previously described [3]. In further support of this notion, the cluster also lacks expression of the marker of cytotoxic lymphoid cells (*NKG7*), that otherwise also express *FCGR3A*. A minor subpopulation expressing *PECAM1* at low levels is present in cluster C12 (0.18% of all CD31+ cells). This cluster appears to be neutrophils, given that most of the cells express *S100A8*, *S100A9, CXCL8* (encoding IL-8), and *CSF3R* [5-8]. Subsets of cells in C12 further express *S100A12* [9], *FCGR3B*, and *CMTM2,* which have all been reported to mark neutrophils as well [8].

The remaining IC clusters (C14-C24) only have cells with low levels of *PECAM1*-expression, which generally represent minor subsets; Based on their expression of *CSF1R, CD14*, *CD74*, *AIF1* among others*,* Clusters C14-C18 and C24 (7.79% and 0.61% of all CD31+ cells, respectively) were ascribed as subtypes of monocytes/macrophages (Figure 6f and Supplementary Figure 6a). Cluster C19 cells (0.14% of all CD31+ cells) appear to be conventional dendritic cells type 1 (cDC1) based on their expression of *CD74* (Figure 6f) and *HLA-D* genes (not shown) at high levels together with previously reported cDC1-markers [3,10] (Supplementary Figure 6b). T cells could be allocated to Clusters C20-C21 (2.99% and 1.64% of all CD31+ cells, respectively) that both contain T cell subtypes expressing the pan-T cell marker *CD3D*. Cluster C20 further has expression of *CD8A* together with markers of cytotoxic- and natural killer [11] cells, indicating that this cluster is a mixture hereof (Supplementary Figure 6b). Cluster C22 (1.68% of all CD31+ cells) represents mast cells expressing *CTSG*, *CMA1*, and *TPSB2* [12,13], and finally, cluster C23 (1.17% of all CD31+ cells) are B cells expressing *CD79A*, *JCHAIN*, and *IGKC* (Supplementary Figure 6b).

8.77% of the CD31+ enriched cells were identified as perivascular mural cells (PC)s that grouped into three clusters (Clusters C25-C27) expressing *RGS5*, *ACTA2*, and *TAGLN*, as well as additional PC subtype markers, but not *PECAM1*. Due to their expression of *PDGFRB*, *ABCC9*, *CSPG4* clusters C25

and C26 appear to be enriched with pericytes, while cluster C27 represents vascular smooth muscle cells (Figure 6d-e and Supplementary Figure 6a).

Finally, 15.72% of the CD31+ cells belong to the adipose stem and progenitor cells (ACs, Clusters C28-C31) expressing the pan-markers of ACs *CFD*, *PDGFRA*, and *DCN* (Figure 6d). Based on the expression of *DPP4*, *CD55*, and *SFRP4*, which are markers of adipose stem cells [14], Cluster C31 (1.35% of all CD31+ cells) appears to contain the most stem-like cells (Supplementary Figure 6b). Notably, the vast majority of cells in this cluster do not express *PECAM1*, which is in contrast to cluster C29 (3.64% of all CD31+ cells), that contains a large subset of cells expressing *PECAM1* at low to moderate levels (Figure 6B, 6E).

Supplementary References

1 Hume DA, Irvine KM, Pridans C. The Mononuclear Phagocyte System: The Relationship between Monocytes and Macrophages. Trends Immunol 2019;40(2):98-112.

2 Elizondo DM, Brandy NZD, da Silva RLL et al. Allograft Inflammatory Factor-1 Governs Hematopoietic Stem Cell Differentiation Into cDC1 and Monocyte-Derived Dendritic Cells Through IRF8 and RelB in vitro [in eng]. Front Immunol 2019;10:173.

3 Hildreth AD, Ma F, Wong YY et al. Single-cell sequencing of human white adipose tissue identifies new cell states in health and obesity. Nat Immunol 2021;22(5):639-653.

4 Baker M, Robinson SD, Lechertier T et al. Use of the mouse aortic ring assay to study angiogenesis. Nat Protoc 2011;7(1):89-104.

5 Yang J, Anholts J, Kolbe U et al. Calcium-Binding Proteins S100A8 and S100A9: Investigation of Their Immune Regulatory Effect in Myeloid Cells. Int J Mol Sci 2018;19(7).

6 Pruenster M, Vogl T, Roth J et al. S100A8/A9: From basic science to clinical application. Pharmacol Ther 2016;167:120-131.

7 Varricchi G, Modestino L, Poto R et al. Neutrophil extracellular traps and neutrophil-derived mediators as possible biomarkers in bronchial asthma. Clin Exp Med 2021.

8 Mistry P, Nakabo S, O'Neil L et al. Transcriptomic, epigenetic, and functional analyses implicate neutrophil diversity in the pathogenesis of systemic lupus erythematosus. Proc Natl Acad Sci U S A 2019;116(50):25222-25228.

9 Pietzsch J, Hoppmann S. Human S100A12: a novel key player in inflammation? Amino Acids 2009;36(3):381-389.

10 Collin M, Bigley V. Human dendritic cell subsets: an update. Immunology 2018;154(1):3-20.

11 Rehman J, Traktuev D, Li JL et al. Secretion of angiogenic and antiapoptotic factors by human adipose stromal cells [in English]. Circulation 2004;109(10):1292-1298.

12 Goldstein N, Kezerle Y, Gepner Y et al. Higher Mast Cell Accumulation in Human Adipose Tissues Defines Clinically Favorable Obesity Sub-Phenotypes. Cells 2020;9(6).

13 Vieira Braga FA, Kar G, Berg M et al. A cellular census of human lungs identifies novel cell states in health and in asthma. Nat Med 2019;25(7):1153-1163.

14 Ferrero R, Rainer P, Deplancke B. Toward a Consensus View of Mammalian Adipocyte Stem and Progenitor Cell Heterogeneity. Trends Cell Biol 2020;30(12):937-950.
